# A Comprehensive Survey of Immune Cytolytic Activity-Associated Gene Co-Expression Networks across 17 Tumor and Normal Tissue Types

**DOI:** 10.3390/cancers10090307

**Published:** 2018-09-04

**Authors:** Tian Tian, Ji Wan, Yan Han, Haoran Liu, Feng Gao, Youdong Pan, Qi Song, Zhi Wei

**Affiliations:** 1 Department of Computer Science, New Jersey Institute of Technology, Newark, NJ 07102, USA; tt72@njit.edu (T.T.); hl425@njit.edu (H.L.); 2 CuraCloud Corporation, Seattle, WA 98104, USA; jiw@curacloudcorp.com (J.W.); yanh@curacloudcorp.com (Y.H.); fengg@curacloudcorp.com (F.G.); youdongp@curacloudcorp.com (Y.P.)

**Keywords:** cytolytic immune activity, tumor immunity, gene co-expression network, system biology

## Abstract

Cytolytic immune activity in solid tissue can be quantified by transcript levels of two genes, *GZMA* and *PRF1*, which is named the CYT score. A previous study has investigated the molecular and genetic properties of tumors associated CYT, but a systematic exploration of how co-expression networks across different tumors are shaped by anti-tumor immunity is lacking. Here, we examined the connectivity and biological themes of CYT-associated modules in gene co-expression networks of 14 tumor and 3 matched normal tissues constructed from the RNA-Seq data of the “The Cancer Genome Atlas” project. We first found that tumors networks have more diverse CYT-correlated modules than normal networks. We next identified and investigated tissue-specific CYT-associated modules across 14 tumor types. Finally, a common CYT-associated network across 14 tumor types was constructed. Two common modules have mixed signs of correlation with CYT in different tumors. Given the tumors and normal tissues surveyed, our study presents a systematic view of the regulation of cytolytic immune activity across multiple tumor tissues.

## 1. Introduction

Immune checkpoint blockade therapy has been proved successful in diverse tumors [1,2], but some fractions of patients still fail to respond [3,4]. Efforts have been made to illuminate the tumor-immune interactions [5] and provide clinical outcome predictors [6,7,8,9]. Recently, Rooney et al. proposed to quantify the cytolytic activity (‘CYT’) of the local immune infiltrate by a measure of the expression levels of two genes (*GZMA* and *PRF1*), which are hallmarks of cytotoxic T cells and natural killer cells activation [10]. This measure is called CYT score. They aimed to explore how the genomic landscape of tumor shapes and is shaped by anti-tumor immunity. They focused on identifying genetic and environmental drivers of tumor-associated cytolytic activity and elucidating how this cytolytic activity is selected for genetic resistance in tumors. Specifically, their results suggested that neoantigens, cancer testis (CT) antigens and viruses are the potential drivers of cytolytic activity. They observed that tumors adapt to cytolytic immune attack by enriching somatic genetic alterations that render them resistant to immune attack. These genetic alterations can be divided into two subsets. One subset of genetic alterations would enable tumors to evade killing but does not impact the infiltration. These alterations include the mutations in antigen presentation machinery genes such as *HLA* and *B2M*, and extrinsic apoptosis genes such as *CASP8*. They also include amplifications in regions of genes that function in immunosuppression such as *PDL1/2*. This subset of alterations is positively correlated with CYT. The other subset of genetic alterations would suppress the immune infiltration and is negatively correlated with CYT. These alterations include the mutations in *IDO1*, *IDO2*, *p53* and *ALOX* gene loci. All these interesting findings suggest that using CYT as trait may allow us to reveal molecular and genetic properties of tumors associated with the local immune cytolytic activity. However, we note that their study was conducted only at single-gene and single-mutation levels. An investigation of the properties and the behaviors of immune cytolytic activity associated gene signatures across different types of tumors at the systems level is lacking.

The past decade has witnessed great advances in high-throughput technologies and network approaches have become a promising method to unravel the system-level properties of gene activities in complex diseases [11,12,13,14,15]. A gene co-expression network is a network based on correlations of gene expression levels [16,17]. In these networks, groups of genes, which are highly correlated in their expressions, are clustered into modules. These modules can then be linked to external traits [18,19] in order to identify trait-specific modules and functions. Furthermore, topological preservations of connectivity of these modules across different networks can be measured, which allows revealing conserved and specific network connections [20]. Highly correlated genes in one module are often thought to reflect functional relationships [21,22], so trait-associated modules across networks of different tissues can provide functional interpretations from the systems biology’s point of view. Compared to other types of biological networks, a gene co-expression network has advantages such that it can cover nearly the complete human transcriptome and does not rely on knowledge obtained from published literature.

By using the RNA-Seq data from the “The Cancer Genomic Atlas” (TCGA) project, we investigated CYT-associated co-expression networks of 17 tumor and normal tissue types. These tissue types are: lung adenocarcinoma (LUAD), lung squamous cell carcinoma (LUSC), kidney renal clear cell carcinoma (KIRC), kidney renal papillary cell carcinoma (KIRP), breast invasive carcinoma (BRCA), colon adenocarcinoma (COAD), stomach adenocarcinoma (STAD), head and neck squamous cell carcinoma (HNSC), liver hepatocellular carcinoma (LIHC), prostate adenocarcinoma (PRAD), thyroid carcinoma (THCA), skin cutaneous melanoma (SKCM), glioblastoma multiforme (GBM), ovarian serous cystadenocarcinoma (OV), and normal lung, breast and kidney (Supplementary Table S1). Here we set out to address three cytolytic immune activity relevant questions by interrogating the CYT-associated co-expression networks. First, are there any differences of CYT-associated modules between tumor and normal networks? Second, do tumors have tumor-specific CYT-associated modules, and if so, what are the functional themes of these specific modules? Third, do different tumors have a common CYT-associated sub co-expression network and what are its functional themes? To answer these three questions, we followed a systematic method of gene co-expression network analysis [15,23,24,25]: first we identified the modules significantly associated with CYT scores; then we interpreted the biological meanings of the identified modules by using pathway enrichment analysis; finally, we summarized the specific and common CYT-associated modules across different tissues. From systems biology’s point of view, our network analyses aim to provide a comprehensive survey of immune cytolytic activity-associated gene signatures.

## 2. Result

### 2.1. Gene Co-Expression Network Construction for Tumor and Normal Tissues

**Figure 1:**
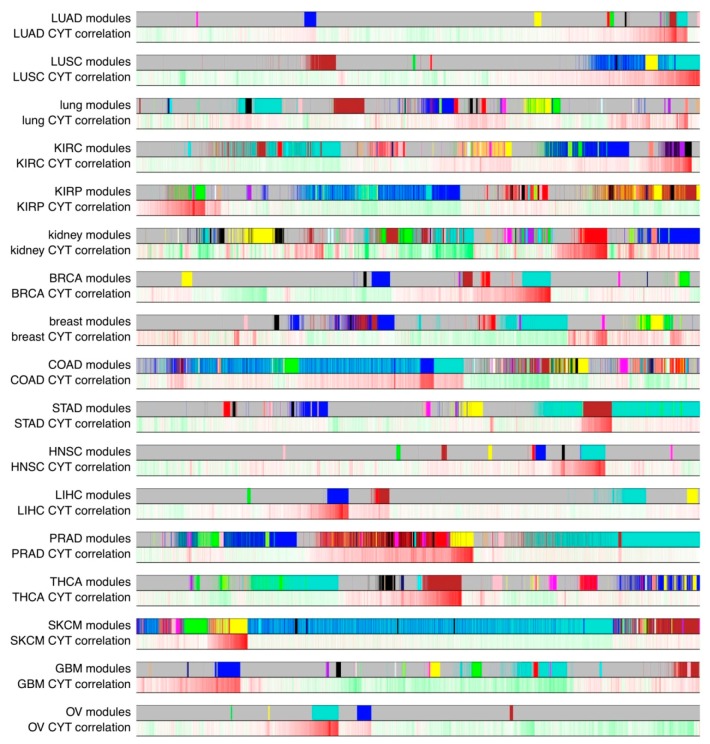
Modules defined in gene co-expression networks and corresponding gene-CYT (cytolytic immune activity) correlations. In each paired two bands, the upper colorful band represents modules in the network, with the largest module in turquoise, second largest in blue, then brown, green, yellow and so on. Grey is not a module, which represents un-grouped genes. The lower band represents the Pearson correlation between genes and CYT scores, red is positively correlated, and green is negatively correlated.

In this study, we analyzed 17 types of tumor and normal tissues with large sample sizes (>100), which are LUAD, LUSC, lung, KIRC, KIRP, kidney, BRCA, breast, COAD, STAD, HNSC, LIHC, PRAD, THCA, SKCM, GBM, and OV. For these data sets, we filtered out genes which had low expression levels, and constructed co-expression network for each tissue type under the protocol of WGCNA (Weighted Gene Co-expression Network Analysis) [17,18,19] based on these selected genes (method). As a result, we constructed 17 tissues-specific co-expression networks containing the same set of 15,677 genes (nodes). These co-expression networks are weighted networks, so any two nodes are connected with an edge weight (from 0 to 1). Modules were defined as a cluster of highly connected genes (nodes) and were considered as biologically related. We plotted colorful bands which represent modules and bands of gene-CYT correlations (Figure 1). We hypothesized that tumor and normal networks might have different characteristics. To further investigate the properties of CYT-associated co-expression networks, we summarized biology themes and connectivity preservations of common and specific modules in tumor and normal networks. The somatic mutation burden, cancer stage, and survival of these CYT-associated modules were also summarized (Supplementary Figures S2–S4). We observed that the genes in CYT-associated modules tended to have higher somatic mutation burdens in most tumors. Many CYT-associated modules were observed to be correlated with cancer stages and survival too.

### 2.2. Tumor Networks Have More Diverse Cytolytic Immune Activity (CYT)-Associated Gene Signatures than Normal Networks

To examine the commonality and differences between tumor and normal networks, we studied CYT-associated modules in three types of tissues which have enough sample sizes (>100) of the tumor and corresponding normal tissues: LUAD and LUSC vs. lung, BRCA vs. breast, KIRC and KIRP vs. kidney.

**Figure 2:**
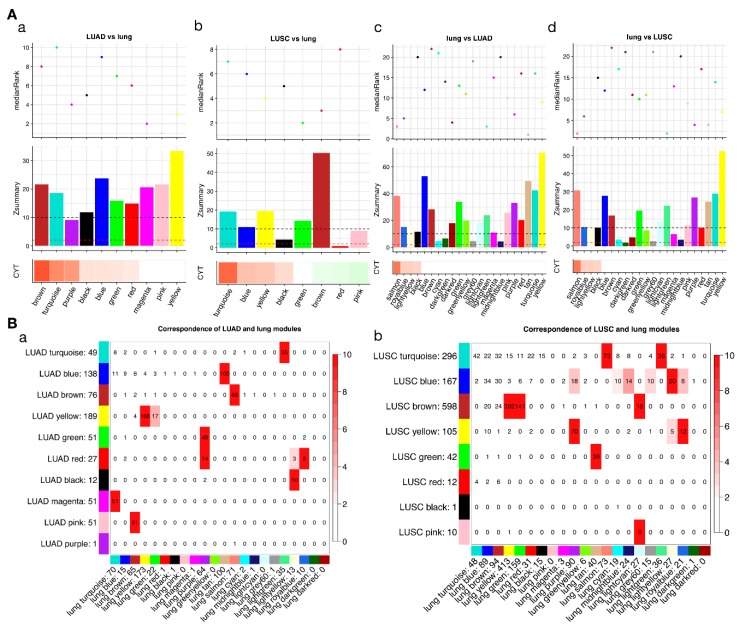
Visualization of CYT-associations and preservations of modules in lung adenocarcinoma (LUAD), lung squamous cell carcinoma (LUSC), and lung. (**A**) In each panel, below band is a plot of module-CYT correlation, with red indicating positively correlated and green indicating negatively correlated. The bar plot in middle shows Z-summary statistics. Red dashed line indicates 2, black dashed line indicates 10. The dot plot in upper shows medianRank statistics. (a) CYT correlation and preservation in lung network of LUAD modules. (b) CYT correlation and preservation in lung network of LUSC modules. (c) CYT correlation and preservation in LUAD network of lung modules. (d) CYT correlation and preservation in LUAD network of lung modules. (**B**) Correspondence of (a) LUAD and (b) LUSC modules and lung modules. Numbers in the table indicate gene counts in the intersection of the corresponding modules. The coloring of the table encodes –log(p), with p being the Fisher’s exact test *p*-value for overlap of two modules.

Correlation analysis of module eigengenes (first principle components of the modules) with CYT scores identified CYT-associated modules. We defined a strong module-trait correlation as that absolute of correlation coefficients that are not less than 0.1 and adjusted *p*-values that are not greater than 0.1 (false discovery rate (FDR) ≤ 0.1). As a result, we found varied CYT-associated modules in LUAD, LUSC, and lung (Figure 2), and these modules were annotated by different biological themes (Table 1). The preservations of connectivity were summarized by Z-summary and medianRank, which were referred to one another [20] (Figure 2A). To see the overlap between modules from tumor and cancer networks, we highlighted the significant levels of overlapping (Figure 2B). The corresponding modules can be identified by significant overlapping (overlapping −log10 (*p*-value) > 2). The gene ontology (GO) themes of common modules across 3 networks were summarized (Supplementary Material). When compared to the lung network, we found LUAD and LUSC had more diverse CYT-associated modules, namely having no corresponding modules in the lung network or having them, but not significantly associated with CYT. These extra CYT-associated modules were themed by “defense response” (“LUAD: turquoise”, corresponding to “lung: lightgreen”, “LUAD: purple”, no corresponding module in lung), “extracellular matrix” (“LUAD: green”, corresponding to “lung: purple”), “vasculature development” (“LUAD: black”, “LUSC: blue”, “lung: lightyellow”), “chromosome segregation” (“LUAD: blue”, corresponding to “lung: tan”), “lamellar body” (“LUSC: black”, no corresponding module in lung), “mRNA processing” (“LUSC: brown”, corresponding to “lung: yellow”, “lung: green”, and “lung: lightcyan”), “oxidoreductase activity” (“LUSC: red”, no corresponding module in lung), “nucleoplasm” (“LUSC: pink”, corresponding to “lung: lightcyan”). These observations suggest that compared to the normal lung, LUAD and LUSC had different gene signatures associated with the immune cytolytic activity, especially tumor networks that had more diverse CYT-correlated gene signatures (tumor CYT-associated modules have more diverse functional themes). To rule out the possibility that missing corresponding modules in the lung was due to the module detection procedure, we further surveyed low preserved modules which had a small Z-summary statistic. Most CYT-associated modules show strong preservation (Z-summary > 10) if we compare the tumor to normal networks. Interestingly, we found “LUSC: red” module was not preserved and did not have the corresponding module in the lung network. “LUSC: red” module was enriched by genes from the “oxidoreductase activity” pathway and negatively correlated with CYT, which was evidence that LUSC has different oxidation metabolic regulation from lung. Oxidoreductase activity is known to be dysregulated in tumors [26], and serves as scavenging reactive oxygen species (ROS) which can promote immunity [27]. So, a negative correlation between oxidoreductase activity module and cytolytic immune activity is not surprising. We illustrated a dysregulation of this module in LUSC by showing its different connectivity property when compared to the normal lung network, which indicated that in LUSC oxidoreductase activity was activated abnormally (Supplementary Figure S2). We also found some modules only shown moderate preservation (2 < Z-summary < 10) such as “LUAD: purple”, “LUSC: black”. “LUSC: black” themed “lamellar body” and was positively correlated with CYT. Lamellar bodies are secretory organelles in type II alveolar cells which are squamous in the lung. They store and release pulmonary surfactant into the extracellular space [28]. Pulmonary surfactant is a lipoprotein complex that plays a role in host defense against infection and inflammation [29]. So, a positive correlation of the module themed by “lamellar body” with cytolytic immune activity is reasonable. Meanwhile, “LUSC: black” was also observed to be correlated with cancer stages (Supplementary Figure S3). Visualization of “LUSC: red” and “LUSC: black” in normal and tumor lung tissues concluded that these genes are highly co-expressed in LUSC only (Supplementary Figure S5).

Next, we compared KIRC, KIRP, and kidney networks (Supplementary Figure S6, Table 2). Kidney, KIRC, and KIRP had common CYT-associated modules (Supplementary Figure S6B, Supplementary Material). Interestingly, modules themed by “mitochondrion” were negatively correlated with CYT in the kidney (“kidney: lightcyan”) but positively correlated with CYT in KIRC (“KIRC: yellow”). We also found KIRC and KIRP had more diverse CYT-associated modules than the kidney (Supplementary Material). In terms of connectivity preservation, we found some CYT-associated modules in the kidney network were not preserved in KIRC and KIRP: “kidney: greenyellow” (themed by “cell periphery” and positively correlated with CYT), “kidney: salmon” (themed by “nephron development” and positively correlated with CYT). An association between glomerular disease membranous nephropathy and malignancy has long been appreciated, and occasional findings of tumor antigens within glomerular immune deposits are supportive of this association [30]. Our network analysis also supported positive correlations between immune activity and cell periphery and nephron development in the kidney, and confirmed KIRC and KIRP networks had such modules disturbed which suggests dysregulation of these two pathways. Visualization of “kidney: greenyellow” and “kidney:salmon” in normal and tumor kidney tissues suggested these genes are co-expressed in kidney only (Supplementary Figure S7). Meanwhile “kidney: darkred” (theme “mitochondrial envelope”) and “kidney: turquoise” (theme “nucleosome”) showed relatively low preservation in KIRC and KIRP networks.

When comparing BRCA with breast networks, again, we found common and unique modules in BRCA and the breast (Supplementary Figure S8, Table 3). The common CYT-associated modules in both breast and BRCA networks were observed (Supplementary Figure S8B, Supplementary Material). In the breast network, we identified the “breast: red” module which themed “Sin3 complex” was negatively correlated with CYT, but its corresponding module in BRCA—“BRCA: yellow” was positively correlated with CYT. We found the genes from the “breast: red” module co-expressed with genes from chromosome segregation pathway in BRCA (“BRCA: yellow”). Sin3 complex is a part of the chromatin-modification machinery that regulates the cell cycle, proliferation, differentiation and cancer pathogenesis. The role of the Sin3 complex in tumorigenesis is still open for debate [31]. Our observation of opposite correlation suggests a role of the Sin3 complex in breast cancer progression. In the BRCA network, we found more diverse CYT-associated modules than in the breast network (Supplementary Material). These CYT-associated modules were preserved in the BRCA and breast networks, although some modules only had moderate preservation (Supplementary Figure S8A, 2 < Z-summary < 10).

In summary, we concluded that although tumor and normal networks have common CYT-associated gene signatures, tumor networks usually had more diverse CYT positively correlated modules than normal networks. Modules themed by “chromosome segregation” and “vasculature development” were correlated with CYT in tumor networks but no significant correlation was detected in normal networks (LUAD, LUSC vs. lung, BRCA vs. breast). Modules themed by “defense response”, “extracellular matrix” contain more genes in tumor networks than normal networks. These results suggested that CYT was a good measure of cytolytic immune activity in both tumor and normal tissues, since we found highly preserved CYT-correlated modules themed by “defense response” in all networks. The results also revealed stronger inflammatory respondes in tumors than in normal tissues, because more diverse co-expressed gene signatures existed in CYT-associated modules of tumors. During tumorigenesis, inflammation activates tissue repair responses and induces proliferation of premalignant cells. Inflammation also stimulates angiogenesis. Angiogenesis promotes the formation of a hospitable microenvironment in which premalignant cells can survive and expand [32]. Meanwhile, adaptive immunity relies on the ability of cytotoxic T cells that need the help of the extracellular matrix. The extracellular matrix can aid recruitment of cellular components of the innate immune system and modify the activation state of the recruited innate immune cells [33]. Our analysis supported that CYT were positively correlated with these pathways.

### 2.3. Some CYT-Associated Modules Are Specific to Tumor Types

**Figure 3:**
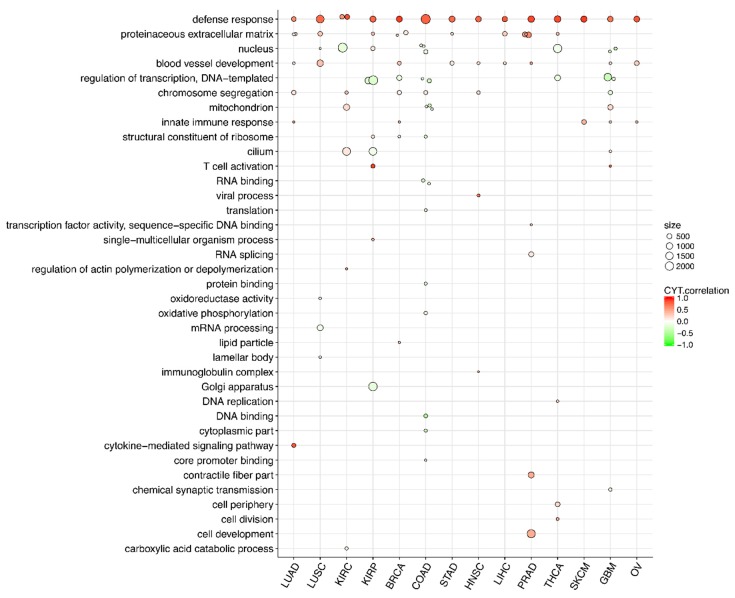
Visualization of gene ontology analysis of CYT-associated modules in 14 types of tumors. Each dot represents a CYT-associated module, with color indicating CYT correlation strength. Dot size is proportional to module size. The x-axis denotes tumor type, and the y-axis denotes representative GO term.

**Figure 4:**
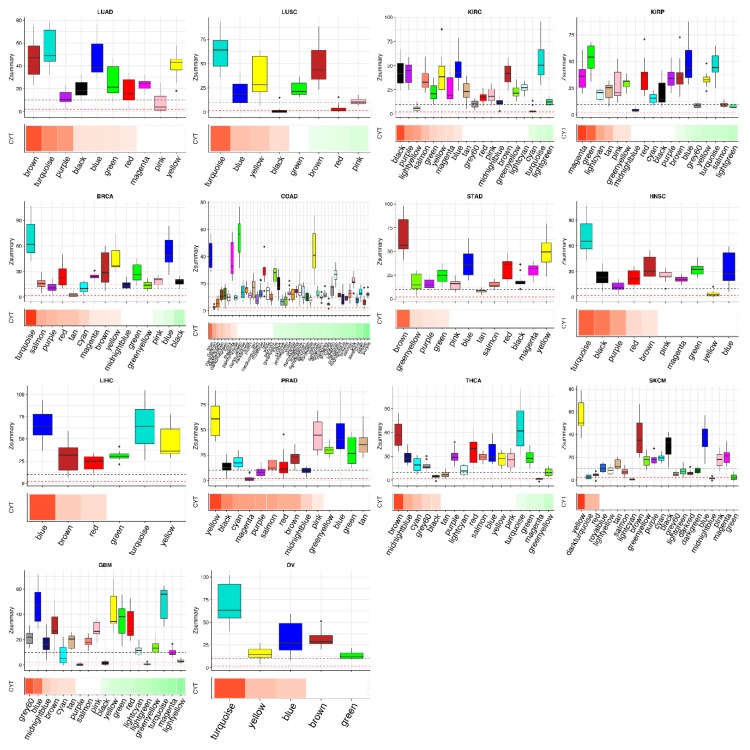
Visualization of CYT-associations and preservations of modules across 14 types of tumors. In each plot, below the band is the module-CYT correlation, with red indicating positively correlated and green for negatively correlated. Upper boxplot is Z-summary statistics of corresponding modules in other 13 tumor networks. Red dashed line indicates 2, black dashed line indicates 10.

Since tumor-specific modules may shed light on specific properties of immunity regulation in each tumor type, we summarized tumor-type-specific CYT-associated modules. We annotated biological themes of CYT-associated modules (Figure 3, Supplementary Table S2). Next, we evaluated the preservation statistics of each module in the contexts of 13 tumor networks other than its own network; thus, we generated 13 Z-summary values for each module (Figure 4). We identified tumor-type-specific CYT module if a CYT-associated module had a median of Z-summary statistics < 2, and moderate conserved CYT module if a CYT-associated module had a median of Z-summary between 2 and 10 (Figure 4). If a module had a median of Z-summary statistics > 10, we defined this module as a highly conserved module.

We found four tumor type-specific modules and annotated them with GO terms (Table 4). These CYT-associated modules can be considered as highly specific in one tumor network since the major Z-summary statistics were below the thresholds of any evidence of preservation in other tumor networks (median Z-summary < 2). The GO themes of these modules were “lamellar body” (LUSC: black) and “oxidoreductase activity” (“LUSC: red”) in LUSC; “extracellular matrix” (“PRAD: magenata”) in PRAD; “chemical synaptic transmission” (“GBM: black”) in GBM. We had already identified “LUSC: black” and “LUSC: red” as specific modules when compared to the normal lung network. Here, we found that these two modules were specific too when compared to all tumor networks. We concluded that the modules themed “lamellar body” and “oxidoreductase” were the specific CYT-associated modules that only exist in LUSC (Supplementary Figures S9 and S10). Intricate roles of the extracellular matrix are important in the transformation from a normal to a malignant cell. The hub genes in the “PRAD: magenta” module were identified to be abnormally expressed in prostate cancers, such as *LAMB3* and *ST6GALNAC2* [34]. Our analysis showed that PRAD had unique properties of regulation of extracellular matrix and immunity compared to other tumors except for SKCM (Z-summary of “PRAD: magenta” in SKCM was 8.1, Figure 4, Supplementary Figure S11). The GBM network has a unique CYT negatively correlated module themed by “chemical synaptic transmission” (“GBM: black”) (Supplementary Figure S12). We also observed “GBM:black” was correlated with survival time (Supplementary Figure S4). Neuro and immune synapses share a common functional unit—phosphatase micro-exclusion, which allows integration of neuroimmune synapses. Neuroimmune synapses control the inflammatory reflex which links vagus nerve activity to inhibition of inflammatory activity [35]. Our analysis also supports a negative correlation between immune activity and synaptic transmission.

We also found some moderately preserved modules across tumor types (Supplementary Table S3), for example, modules themed by “carboxylic acid catabolic process” in KIRC (“KIRC: cyan”) and “lipid particle” in BRCA (“BRCA: tan”), etc. Other CYT-associated modules can be considered as highly preserved across different tumors in terms of the properties of network connectivity.

### 2.4. Different Tumors Have a Common CYT-Associated Sub-Network

We observed that many CYT-associated modules were highly topologically preserved (Figure 4) and shared similar biological themes (Figure 3). These observations suggested common properties of CYT-associated co-expression networks. The common representative GO themes across 14 tumors, divided into three groups based on their CYT association directions, included: (1) positively CYT-associated ones, such as “defense response”, “proteinaceous extracellular matrix”, “blood vessel development” and “chromosome segregation” (except for “GBM: yellow”); (2) negatively CYT-associated ones, such as “nucleus” and “regulation of transcription”; and (3) mixed CYT-associated ones, such as “mitochondrion”, “cilium” and “structural constituent of ribosome”.

**Figure 5:**
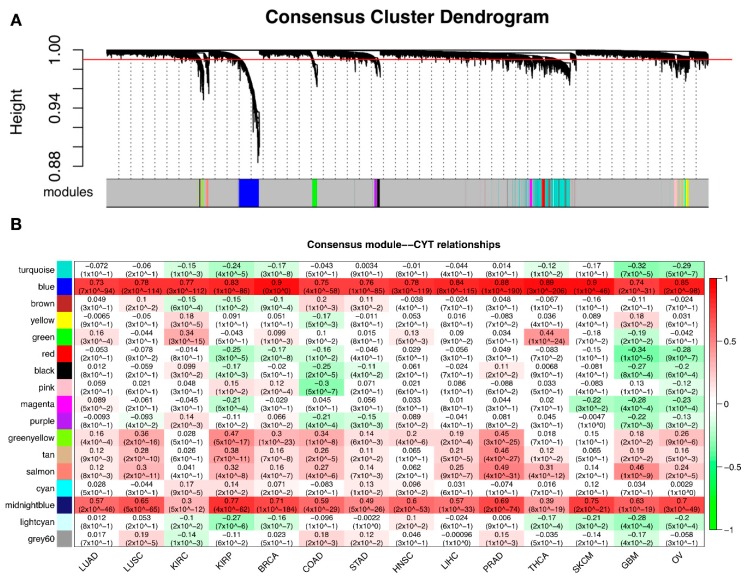
Consensus network of 14 tumor sets. (**A**) Dendrogram of the consensus network. Consensus modules are marked by colors on the horizontal bar. (**B**) Matrix with modules-CYT correlations and corresponding adjusted *p*-values between consensus modules in y-axis and tumor types in x-axis. Green for negative correlation, red for positive correlation.

Based on the observation of common CYT-associated modules across different tumor types, we hypothesized that tumors had a common CYT-associated co-expression sub-network. For this purpose, we constructed a consensus co-expression network based on 25% percentile of TOMs (topology overlay matrix) of 14 tumor networks. We identified modules in this consensus network and correlated these consensus modules with CYT scores in different tumor types (Figure 5). To identify biology themes of these consensus modules, we conducted gene ontology analysis (Table 5). We observed that consensus module “blue” themed “defense response” was highly positively correlated with CYT scores in all tumor types. Consensus modules “green”, “greenyellow”, “tan” and “salmon” were positively correlated with CYT scores in most tumor types, and were themed with “chromosome segregation”, “cell periphery”, “circulatory system development” and “proteinaceous extracellular matrix” respectively. In the GBM network, consensus module “green” (“chromosome segregation”) was negatively correlated with CYT, which was consistent with the result of individual GBM network (“GBM: yellow”, Supplementary Figure S12). Consensus modules “turquoise”, “red”, “black” and “magenta” themed with “regulation of transcription”, “regulation of gene expression”, “RNA splicing” and “nuclear lumen”, respectively, were negatively correlated with CYT scores in most tumor types. Module “yellow” and “pink” themed with “mitochondrial respiratory chain complex assembly” (KIRC, COAD, GBM) and “structural constituent of ribosome” (KIRP, BRCA, COAD) had mixed signs of correlation with CYT scores. These results were consistent with the analysis of CYT-associated modules in individual tumor networks (Figure 3).

In summary, we concluded that there was a common CYT-associated co-expression sub-network across different tumor types, and these consensus modules can be divided into three groups: positively, negatively, and mixedly correlated with CYT scores. Specifically, CYT positively correlated modules were enriched with genes in categories of “defense response”, “proteinaceous extracellular matrix”, “blood vessel development” and “chromosome segregation”. The pathway “defense response” appears reasonable since CYT score is a measure of local cytolytic immune activity. The enriched themes were well-known regulation processes of immunity, which supports our analysis results well. CYT negatively correlated modules were enriched with genes in categories of “regulation of transcription”, “regulation of gene expression”, “RNA splicing” and “nucleus”. Of note is “regulation of transcription”. High CYT means a high local cytolytic immune activity that may suppress the transcription activity in tumor tissues.

Common modules themed by “structural constituent of ribosome” and “mitochondrial” had mixed CYT correlations. Many studies have proved important roles of ribosome and mitochondria in immune signaling and tumorigenesis [36,37,38]. We found that CYT-associated modules themed commonly by “cilium” in KIRC, KIRP, and GBM were not presented in the consensus network. Cytotoxic T lymphocytes (CTLs) kill tumor cells by forming a cytolytic synapse with their target cell. Marked reorganization of both the actin and the microtubule cytoskeletons brings the centrosome up to the plasma membrane to the point of T cell receptor signaling. Such centrosomal docking also occurs during ciliogenesis. Actually, the formation of the CTL synapse and ciliogenesis shares common molecular machinery such as the hedgehog pathway [39]. So, a correlation between modules themed by the “cilium” pathway and cytolytic immune activity is expected. However, we observed a negative correlation between cilium module and CYT in KIRP, which needs further study. This analysis depicted a common cytolytic immunity-associated co-expression network across different tumor types, and revealed unique CYT properties in some tumors.

## 3. Discussion

In this study, we built gene co-expression networks of the tumor and normal tissues and identified common and specific properties of these networks. This WGCNA approach reduces various confounding factors in data analysis which affects other types of biological networks such as batch effects, and does not rely on the bias of prior knowledge. We summarized CYT-associated co-expression modules and compared properties of these modules in different tissues. Specifically, we came to three major conclusions. First, tumor and normal networks have different CYT-associated gene signatures. Second, some tumor networks have specific CYT-associated modules in terms of connectivity patterns, and pathway analysis reveals the biological functions of these specific modules. Third, different tumor networks have a common CYT-associated sub-network. To our best knowledge, this is the first systematic survey of gene signatures of cytolytic immune activity across various tumors of data from clinical patients. These findings give us hints to understand regulations of immune activity in different tumors and why tumors have varied responses to immune therapy.

It is not surprising that tumor and normal tissues have different gene signatures associated with the cytolytic immune activity. We found that in tumor networks more genes of immunity pathway (larger co-expression modules themed by “defense response”) were correlated with CYT scores than in normal networks, which is plausible since tumorigenesis can arouse inflammation and activate more immune response genes [32]. Notably, compared to normal tissues, we identified tumor networks have common CYT positively correlated modules which were themed by “chromosome segregation” and “vasculature development”, but these modules were not always correlated with CYT in normal networks. This observation is consistent with the known role of immunity in tumorigenesis [32]. We did not rely on any prior knowledge to identify these modules, which proved that our system approach could reveal established mechanisms of immune regulation in tumorigenesis. We also found that these two kinds of modules (“chromosome segregation” and “vasculature development”) were positively correlated with CYT scores across five and nine tumors respectively, which represents a universal common regulation of immunity in different tumor tissues.

It is noted that the normal samples from TCGA were collected from the same cancer patients. They are the so-called matched controls in clinical studies. Using matched controls will help to reduce confounding impacts brought by individual variances, but it is worth noting that these samples are known to exhibit cancerous signatures sometimes. Alternatively, we may consider using normal tissues from healthy individuals (un-matched controls), e.g., data from the genotype-tissue expression project (GTEx) study, for comparison. However, integrative analysis of genomic data across different studies poses great challenges and appropriate methods correcting for study-specific biases are under development [40]. We leave the comparison with un-matched controls to our future research.

Across different tumor co-expression networks, we identified specific and common CYT-associated modules based on connectivity properties. Common modules suggested a conserved regulation network of cytolytic immune activity in different tumor tissues. These common modules were supported by our current knowledge of the role of immune system in tumorigenesis [32,33,41,42]. Specific modules revealed specific regulation of cytolytic immune activity in one tissue and these unique properties can be further studied. The unique gene signatures of local cytolytic immune activity revealed the diversity of immunity regulation in different tumor tissues and may be the key to understanding the difference of immune response in various tumor tissues. This study provided a full picture of cytolytic immune activity regulation networks in different tumor tissues by showing common and specific co-expression modules linking to immune activity.

In summary, our study provides a system-level insight into the gene signatures of local cytolytic immune activity, which provides evidence of specific and common regulation networks of immunity in different tumor and normal tissues, and gives us hints in understanding mixed immunotherapy responses in different tumors. We identified CYT-associated modules in various tissues. Furthermore, hub genes of each module could serve as candidate biomarkers. Further efforts are required to validate and extend our findings. First, our findings are based on TCGA data, but a lack of normal tissue data in TCGA limited us to do more tumor/normal network comparisons. So, a more comprehensive dataset containing large sample sizes of the tumor and normal tissues is needed, which could help us to find more properties of immunity regulation in tumorigenesis. GTEx [43] is a good source of transcriptome data of normal tissues, but study-specific bias-corrected data which is comparable to TCGA is lacking. The second effort is to identify regulatory networks in various tissues. Gene regulatory networks are directional networks which are based on the Bayesian inference. By incorporating other types of genetic data such as eQTL (expression quantitative trait loci) data, Bayesian networks can infer the direction of gene regulation [44]. Finally, future efforts should be made to confirm this system-level analysis by experimental practice.

## 4. Materials and Methods

### 4.1. Data Processing

We obtained TCGA gene expression data by TCGA-assembler [45]. We downloaded data of the assay Platform “gene.normalized_RNAseq”, which is normalized gene expression quantification (normalized reads per kilobase of transcript per million mapped reads, RPKM data). Based on the TCGA barcodes, we selected primary solid tumor and solid normal tissues only. As a result, we obtained 6416 samples of 17 different tumor and normal tissue types. To reduce background noise, we applied two criteria to filter out low expressed genes across all these samples: the first one selects any gene whose 50th-percentile RPKM value is greater than 1, and the second one selects any gene whose 90th-percentile RPKM value is greater than 10. Next, we cleaned outlier samples in each tissue type based on the expression levels of these highly expressed genes. We followed the methods of outlier labeling based on mean IAC (inter-array correlation) [46]. We first calculated the Pearson correlation coefficient based the expression levels for any paired samples in each tissue set (IACs), then the mean IACs of one sample is defined as the mean of IACs of all pairs that contain this sample. We excluded samples with low mean IACs. Low mean IAC is defined as samples with mean IACs that are lower than mean of mean IACs minus 2 times of standard deviation of mean IACs. After outlier cleaning, we applied another filter of genes which is that the median absolute deviations (MAD) of each gene in each tissue set should be greater than 0. This was required by calculating biweight mid-correlation (bicor) [19]. As a result, we obtained a same set of 15,677 genes (excluding *GZMA* and *PRF1*) in each tissue set (sample sizes and clinical information are summarized in Supplementary Table S1. Since normal tissues are also from the same cancer patients, so we also summarize clinical information of the patients who have normal tissue.). To build the co-expression networks and calculate CYT scores, we transferred RPKM values into transcripts per million (TPM) values by using the formula $TPM_{i}=\left(\frac{RPKM_{i}}{sum\left(RPKM_{j}\right)}\right)\times 10^{6}$ for gene *i*. TPM values were log2 transformed (log2(TPM_i_+1)). Expression levels of *GZMA* and *PRF1* were highly correlated across all samples (Supplementary Figure S1), and CYT scores were defined as log-average of *GZMA* and *PRF1* expression in TPMs.

### 4.2. Co-Expression Network Construction

The gene-CYT correlation was calculated by Pearson correlation. The sign of Pearson correlation defined the sign of gene-CYT correlation. Co-expression networks were constructed by following the procedure of WGCNA [17], in which signed networks and biweight mid-correlation are used. Argument maxPOutlier = 0.1 was set for bicor. Biweight mid-correlation combines advantages of the Pearson correlation (relatively high power) and the Spearman correlation (relatively high robustness). The key parameter, β, was optimized for each individual network so that a good scale-free topology fitting can be achieved (fitting index above 0.8). Genes were grouped by applying complete linkage hierarchical clustering on the topology overlay matrix (TOM). We identified modules by the dynamic hybrid tree cut technique [47], with parameter settings: height cutoff = 0.95, deepSplit = 4 and minimum cluster size = 20, on the resulting dendrogram (we used these settings for filtering out genes that were not so closely correlated and dividing genes into more subtle co-expression modules). The key parameters we used for WGCNA were summarized in Supplementary Table S4. WGCNA may report many false discovered correlations, but the majority of the discovered correlations should be correct. It is noted that the systems biology approaches we used focused on a set of findings and were robust against those potential individual errors. Therefore, we were not concerned about the false discovered correlations, even though there are many but not a majority. Modules eigengenes can be seen as the first principal component of a module. Similar modules with small dissimilarity (1—correlation), which was defined as a maximum cutoff of 0.15, of eigengenes were merged. To identify CYT-associated modules, we correlated module eigengenes with CYT scores. We discarded large modules which had more than 3000 genes, since a large cluster of genes may be artificial (which meant the data did not have enough resolution to differentiate these genes) and vague to identify representative biology themes. Correlation *p*-values were adjusted for multiple comparisons with the Benjamini and Hochberg correction (FDR) [48]. CYT-associated modules were defined as absolute correlation coefficients not less than 0.1 and FDR not greater than 0.1.

### 4.3. Analysis of Somatic Mutation and Clinical Information

Mutation burdens of genes in the CYT-associated modules were summarized. The somatic mutation information was downloaded and processed by TCGA-assembler. For each gene, we summarized the proportion of patients who have somatic mutations. One-sided *t*-test was applied to compare if the mutation proportions were higher in genes from the CYT-associated modules than in non-CYT genes.

Clinical information was downloaded using TCGA-assembler. We used module eigenes to analyze the correlation between modules and clinical information. For cancer stage information, to rule out the unreliability of small sample size, we discarded the stages that have less than 3 patients. We applied the Kruskal–Wallis rank sum to test if there are significant differences of eigene values of each module across stages. For module survival analysis, we first divided patients into two groups: low and high, which was defined by the median of module eigene values. We applied Kaplan–Meier survival analysis [49] to test if there is significant difference of the survival rates between low and high groups. The significance threshold is set as *p*-value < 0.05.

### 4.4. Module Preservation Statistics

The WGCNA package provides a statistical test for module preservation analysis [20]. We calculated 200 permutations of these preservation statistics to generated Z-summary and medianRank statistics. The Z-summary indicates the preservation of network topology of modules, with the thresholds of strong preservation (Z-summary > 10), moderate preservation (2 < Z-summary < 10) and not preserved (Z-summary < 2). Z-summary is a composite statistic of density and connectivity measures but is sensitive to module size. So, we also calculated medianRank. The medianRank statistics are based on observed preservation statistics but is less dependent on module size. We used medianRank as a measure of relative preservation of modules with different sizes, lower ranks indicating more preservation.

### 4.5. Consensus Network Construction

A consensus network is a common network that exists in all tumor types. We constructed a consensus network following the procedure provided by WGCNA [50]. Since topological overlap matrices of different tumor types may have different statistical properties (constructed by different βs); we first scaled the TOMs of other tumor types such that the 95th percentile equals the 95th percentile of the TOM of LUAD (LUAD was selected by artificial, we made all TOMs comparable to LUAD’s TOM). After scaling, we calculated the consensus topological overlap by taking the tissue-wise 25% percentile of the TOMs of individual sets. Thus, for input networks *A^(^*^1)^, *A*^(2)^, …, *A*^(k)^ and quantile q (we used 25% here), we defined the consensus TOM as:

$$Consensus_{q}\left(A^{\left(1\right)},\;A^{\left(2\right)},\;\ldots ,\;A^{\left(k\right)}\right)=\;Quantile_{q}\left(sTOM^{\left(1\right)},sTOM^{\left(2\right)},\ldots ,sTOM^{\left(k\right)}\right)$$

where *sTOM* is the scaled topological overlap measure. We applied the dynamic hybrid tree cut algorithm to detect modules in the consensus network, with the parameters: height cutoff = 0.99, deepSplit = 4, and minimum cluster size = 20. We merged similar modules (dissimilarity cutoff 0.15) after the module detection procedure.

### 4.6. Gene Ontology Enrichment Analysis

GO enrichment analysis was conducted by the R package GOFunction [51] with default parameters. Basically, GOFunction compares the proportion of genes from the GO terms in the module and the proportion in the background genes (all expressed genes except module genes), and the terms with proportions significantly higher in the module are considered as enriched. GOFunction first selected terms by the significant level of FDR 0.05 (FDR was adjusted by the Benjamini and Yekutieli FDR procedure [52]). For these terms, GOFunction removed local redundancy by comparing between ancestor and offspring with overlapping genes, and global redundancy by comparing between non-ancestor–offspring terms with overlapping genes. Significance levels of all comparisons were set to FDR 0.05 by default. We selected GO terms labeled “Final” as the representative functions of a module, which were significantly enriched terms that remained after removing local and global redundancy. We applied enrichment analysis for either the ontology of “BP” (Biological Process), “CC” (Cellular Component) or “MF” (Molecular Function), and obtained enriched lists of GO terms by combining all three ontologies. The lists were sorted by enrichment *p*-values.

For each module, we chose a fair GO term from the list of terms selected by GOFunction as the representative GO term. Given the redundancy of GO terms, two functional similar modules may be assigned by different GO terms. To reduce this problem, we calculated semantic distances between these GO terms and clustered them. We further chose a GO term as representative GO theme from closely clustered GO terms. This procedure of clustering and selecting representative terms was done by REVIGO (Reduce Visualize Gene Ontology) [53].

## 5. Conclusions

Our study provides a system-level insight into the gene signatures of local cytolytic immune activity. It presents evidence of specific and common regulation networks of immunity in different tumor and normal tissues, and gives hints of understanding of mixed immunotherapy responses in different tumors.

## Figures and Tables

**Table 1:** Gene ontology (GO) analysis of CYT-associated modules in LUAD, LUSC and lung networks.

**LUAD**	**CYT Correlation**	**CYT Adjust *p***	**Size**	**Representative GO**	**Intersect Number**	**Enrichment Adjust *p***
brown	0.824296	2.22 × 10^−145^	261	GO:0019221: cytokine-mediated signaling pathway	45	<2.2 × 10^−16^
turquoise	0.59894496	1.25 × 10^−51^	398	GO:0006952: defense response	141	<2.2 × 10^−16^
purple	0.522830484	7.96 × 10^−37^	25	GO:0002250: adaptive immune response	7	0.017494691
black	0.156820899	0.001366798	53	GO:0001944: vasculature development	20	1.69 × 10^−11^
blue	0.152476114	0.001569748	331	GO:0007059: chromosome segregation	93	<2.2 × 10^−16^
green	0.1433875	0.002702892	103	GO:0005615: extracellular space	54	<2.2 × 10^−16^
red	0.113753405	0.018649506	74	GO:0031012: extracellular matrix	16	5.74 × 10^−6^
**LUSC**	**CYT Correlation**	**CYT Adjust *p***	**Size**	**Representative GO**	**Intersect Number**	**Enrichment Adjust *p***
turquoise	0.747554212	3.95 × 10^−98^	1622	GO:0006952: defense response	432	<2.2 × 10^−16^
blue	0.329301026	3.60 × 10^−13^	999	GO:0001568: blood vessel development	100	<2.2 × 10^−16^
yellow	0.2925106	1.40 × 10^−10^	379	GO:0005615: extracellular space	106	<2.2 × 10^−16^
black	0.189279714	6.43 × 10^−5^	41	GO:0042599: lamellar body	3	0.01760483
brown	−0.10539746	0.023468284	796	GO:0006397: mRNA processing	59	2.36 × 10^−10^
red	−0.136069487	0.003582747	45	GO:0016491: oxidoreductase activity	21	<2.2 × 10^−16^
pink	−0.18133647	0.000111756	23	GO:0005654: nucleoplasm	16	0.011973696
**Lung**	**CYT Correlation**	**CYT Adjust *p***	**Size**	**Representative GO**	**Intersect Number**	**Enrichment adjust *p***
salmon	0.685802999	5.03 × 10^−16^	76	GO:0002250: adaptive immune response	23	<2.2 × 10^−16^
royalblue	0.252541858	0.074263653	29	GO:0030198: extracellular matrix organization	14	0.000755567
black	0.231568485	0.099027542	324	GO:0000139: Golgi membrane	31	2.50 × 10^−2^

**Table 2:** Gene ontology analysis of CYT-associated modules in kidney renal clear cell carcinoma (KIRC), kidney renal papillary cell carcinoma (KIRP) and kidney networks.

**KIRC**	**CYT Correlation**	**CYT Adjust *p***	**Size**	**Representative GO**	**Intersect Number**	**Enrichment Adjust *p***
black	0.853237771	7.92 × 10^−175^	442	GO:0006952: defense response	147	<2.2 × 10^−16^
purple	0.540851383	1.38 × 10^−40^	371	GO:0006952: defense response	123	<2.2 × 10^−16^
light yellow	0.483655412	3.41 × 10^−31^	24	GO:0008064: regulation of actin polymerization or depolymerization	6	0.006282833
salmon	0.334088519	4.48 × 10^−14^	122	GO:0007059: chromosome segregation	55	<2.2 × 10^−16^
yellow	0.195625539	2.79 × 10^−5^	895	GO:0005739: mitochondrion	265	<2.2 × 10^−16^
magenta	0.181397917	9.45 × 10^−5^	378	GO:0005634: nucleus	202	7.71 × 10^−5^
blue	0.132458919	0.005354675	1718	GO:0005929: cilium	70	0.003733141
cyan	−0.149121774	0.001600027	106	GO:0046395: carboxylic acid catabolic process	16	8.63 × 10^−8^
turquoise	−0.190383188	4.25 × 10^−5^	2322	GO:0005654: nucleoplasm	630	<2.2 × 10^−16^
**KIRP**	**CYT Correlation**	**CYT Adjust *p***	**Size**	**Representative GO**	**Intersect Number**	**Enrichment Adjust *p***
magenta	0.890605949	7.90 × 10^−121^	261	GO:0042110: T cell activation	57	<2.2 × 10^−16^
green	0.765114172	3.91 × 10^−61^	810	GO:0006952: defense response	254	<2.2 × 10^−16^
lightcyan	0.455276412	3.39 × 10^−15^	43	GO:0072359: circulatory system development	20	1.52 × 10^−9^
tan	0.351260084	6.85 × 10^−9^	107	GO:0005578: proteinaceous extracellular matrix	21	2.31 × 10^−11^
pink	0.150757415	0.019363345	311	GO:0005654: nucleoplasm	94	0.00567888
green yellow	0.1482446	0.020133545	113	GO:0003735: structural constituent of ribosome	64	<2.2 × 10^−16^
brown	−0.113504399	0.081563375	1623	GO:0005929: cilium	73	0.000130924
blue	−0.17038931	0.007845967	2117	GO:0005794: Golgi apparatus	285	4.72 × 10^−13^
yellow	−0.235236938	0.000162479	1124	GO:0051252: regulation of RNA metabolic process	391	<2.2 × 10^−16^
turquoise	−0.242691756	0.000120124	2258	GO:0006355: regulation of transcription, DNA-templated	651	<2.2 × 10^−16^
**Kidney**	**CYT Correlation**	**CYT Adjust *p***	**Size**	**Representative GO**	**Intersect Number**	**Enrichment Adjust *p***
red	0.901777428	2.39 × 10^−47^	762	GO:0006952: defense response	237	<2.2 × 10^−16^
green yellow	0.642030403	5.42 × 10^−13^	264	GO:0071944: cell periphery	119	1.20 × 10^−7^
dark green	0.43921949	1.18 × 10^−5^	30	GO:0006412: translation	20	< 2.2 × 10^−16^
light green	0.408015885	5.90 × 10^−5^	66	GO:0003735: structural constituent of ribosome	44	<2.2 × 10^−16^
salmon	0.36223561	0.000495518	179	GO:0072006: nephron development	12	9.11 × 10^−5^
lighty ellow	−0.201536545	0.099115723	58	GO:0006355: regulation of transcription, DNA-templated	29	0.003488448
light cyan	−0.323038323	0.002327917	122	GO:0006119: oxidative phosphorylation	35	<2.2 × 10^−16^
dark red	−0.514005641	1.07 × 10^−7^	30	GO:0005740: mitochondrial envelope	11	8.30 × 10^−5^
turquoise	−0.557270749	3.68 × 10^−9^	1462	GO:0000786: nucleosome	17	0.0025

**Table 3:** Gene ontology analysis of CYT-associated modules in breast invasive carcinoma (BRCA) and breast networks.

**BRCA**	**CYT Correlation**	**CYT Adjust *p***	**Size**	**Representative GO**	**Intersect Number**	**Enrichment Adjust *p***
turquoise	0.911502399	0	796	GO:0006952: defense response	292	<2.2 × 10^−16^
salmon	0.420106646	1.23 × 10^−46^	33	GO:0051607: defense response to virus	20	<2.2 × 10^−16^
red	0.325051705	4.20 × 10^−27^	210	GO:0001944: vasculature development	36	<2.2 × 10^−16^
tan	0.316862948	8.28 × 10^−26^	34	GO:0005811: lipid particle	6	9.47 × 10^−5^
cyan	0.242642809	2.68 × 10^−15^	32	GO:0031012: extracellular matrix	9	0.004274631
magenta	0.119753573	0.000159247	55	GO:0003735: structural constituent of ribosome	50	<2.2 × 10^−16^
brown	0.117448707	0.000197181	302	GO:0005615: extracellular space	92	<2.2 × 10^−16^
yellow	0.102251834	0.001113399	287	GO:0007059: chromosome segregation	89	<2.2 × 10^−16^
blue	−0.165492877	1.17 × 10^−7^	523	GO:0010468: regulation of gene expression	226	<2.2 × 10^−16^
**Breast**	**CYT Correlation**	**CYT Adjust *p***	**Size**	**Representative GO**	**Intersect Number**	**Enrichment Adjust *p***
magenta	0.651782549	1.06 × 10^−14^	78	GO:0006955: immune response	50	<2.2 × 10^−16^
tan	0.47330672	6.74 × 10^−7^	28	GO:0001944: vasculature development	9	0.037762588
green yellow	0.359311022	0.000433761	33	GO:0005615: extracellular space	18	5.37 × 10^−9^
red	−0.199648593	0.078699329	255	GO:0016580: Sin3 complex	5	0.022387309
turquoise	−0.215023696	0.062018398	1820	GO:0005634: nucleus	1104	<2.2 × 10^−16^
purple	−0.249189975	0.027500087	66	GO:0016569: regulation of transcription, DNA-templated	35	0.0001246457

**Table 4:** Gene ontology analysis of tumor-type-specific CYT-associated modules. Tumor type-specific is defined as median Z-summary < 2.

Module	CYT Correlation	CYT Adjust *p*	Size	Representative GO	Intersect Number	Enrichment Adjust *p*	Median Z-Summary
LUSC:black	0.189279714	0.0000643	41	GO:0042599: lamellar body	3	0.01760483	0.212845087
LUSC:red	−0.136069487	0.003582747	45	GO:0016491: oxidoreductase activity	21	<2.2 × 10^−16^	1.532464475
Prostate adenocarcinoma (PRAD):magenta	0.492176	1.28 × 10^−30^	427	GO:0005615: extracellular space	49	0.000711	1.263953
Glioblastoma multiforme (GBM):black	−0.142172924	0.096014779	133	GO:0007268: chemical synaptic transmission	40	<2.2 × 10^−16^	1.641635813

**Table 5:** Gene ontology analysis of consensus modules across 14 tumors.

Module	Size	Representative GO ID	Representative GO	Intersect Number	Enrichment Adjust *p*
turquoise	745	GO:0006355	regulation of transcription, DNA-templated	276	<2.2 × 10^−16^
blue	523	GO:0006952	defense response	212	<2.2 × 10^−16^
yellow	113	GO:0033108	mitochondrial respiratory chain complex assembly	19	<2.2 × 10^−16^
green	112	GO:0007059	chromosome segregation	49	<2.2 × 10^−16^
red	89	GO:0010468	regulation of gene expression	48	4.33 × 10^−10^
black	72	GO:0008380	RNA splicing	14	1.79 × 10^−6^
pink	70	GO:0003735	structural constituent of ribosome	62	<2.2 × 10^−16^
magenta	68	GO:0031981	nuclear lumen	49	5.51 × 10^−12^
green yellow	60	GO:0071944	cell periphery	39	1.92 × 10^−6^
tan	58	GO:0072359	circulatory system development	21	1.95 × 10^−7^
salmon	50	GO:0005578	proteinaceous extracellular matrix	26	<2.2 × 10^−16^
cyan	33	GO:0006119	oxidative phosphorylation	8	3.35 × 10^−6^

## Supplementary Materials

The following are available online at http://www.mdpi.com/2072-6694/10/9/307/s1. Figure S1: Correlation (Pearson’s correlation) of expression levels of GZMA and PRF1 across all 6114 samples. Figure S2: Mutation burden of genes in CYT-associated modules. The means (the dots) and standard errors (error bars) and mutation proportion are displayed. Figure S3: Plots of CYT-associated modules in cancer stages. Figure S4: Plots of survival analysis of CYT-associated modules. Figure S5: Visualization of “LUSC: black” and “LUSC: red” modules in LUSC, LUAD and lung networks. Figure S6: Visualization of CYT-associations and preservations of modules in KIRC, KIRP and kidney. Figure S7: Visualization of “kidney: greenyellow” and “kidney: salmon” modules in kidney, KIRC and KIRP networks. Figure S8: Visualization of CYT-associations and preservations of modules in BRCA and breast. Figure S9: Visualization of “LUSC: black” across 14 tumor networks. Figure S10: Visualization of “LUSC: red” across 14 tumor networks. Figure S11: Visualization of “PRAD: magenta” across 14 tumor networks. Figure S12: Visualization of “GBM: black” across 14 tumor networks. Table S1: Summary of TCGA gene expression data used to construct co-expression networks. Table S2: Summary of CYT-associated modules across 14 types of tumors. Table S3: Gene ontology analysis of moderate conserved CYT-associated modules, which are defined as median 2 < Z-summary < 10. Table S4: WGCNA parameters.
