# Supplementary material for: A Comprehensive Survey of Immune Cytolytic Activity-Associated Gene Co-Expression Networks across 17 Tumor and Normal Tissue Types
Source: Cancers (Basel). 2018 Sep 4;10(9):307. doi: 10.3390/cancers10090307 (PMC6162652; doi:10.3390/cancers10090307)
Supplement: Supplementary file 1 [file cancers-10-00307-s001.zip › cancers-332194-supplementary-final-/cancers-332194-supplementary-R2 revised - layout.docx]

Corresponding CYT-Associated Modules in Tumor and Normal Tissues

According to Figure 1 and Table 1, common CYT-associated modules across LUSC, LUAD and lung were themed by “defense response” (“lung: salmon”, “LUAD: brown”, “LUSC: turquoise”), “extracellular matrix” (“lung: royalblue”, “LUAD: red”, “LUSC: yellow”).

According to Figure 2 and Table 2, common CYT-associated modules across KIRC, KIRP and kidney were themed by “defense response” (“kidney: red”, “KIRC: black”, “KIRC: purple”, “KIRP: magenta”, “KIRP: green”), “structural constituent of ribosome” (“kidney: darkgreen”, “kidney: lightgreen”, “KIRP: greenyellow”), “regulation of transcription” (“kidney: lightyellow”, “KIRP: yellow”, “KIRP: turquoise”), “mitochondrion” (“kidney: lightcyan”, “KIRC: yellow”), “nucleosome” (“kidney: turquoise”, “KIRC: turquoise”, “KIRP: turquoise”). Meanwhile, we found KIRC and KIRP have more CYT-associated modules than kidney. They were modules themed by “regulation of actin polymerization or depolymerization” (“KIRC: lightyellow”, corresponding to “kidney: green”), “nucleus” (“KIRC: magenta”, “KIRP: pink”, corresponding to “kidney: green”, “kidney: magenta”, “kidney: purple”, “kidney: cyan”, “kidney: grey60”), “cilium” (“KIRC: blue”, “KIRP: brown”, corresponding to “kidney: green”, “kidney: magenta”, “kidney: tan”), “carboxylic acid catabolic process” (“KIRC: cyan”, corresponding to “kidney: blue”), “Golgi apparatus” (“KIRP: blue” corresponding to “kidney: yellow”, “kidney: black”, “kidney: midnightblue”, “kidney: royalblue”).

According to Figure 3 and Table 3, common CYT-associated modules across BRCA and breast were themed by “defense response” (“breast: magenta”, “BRCA: turquoise”), “vasculature development” (“breast: tan”, “BRCA: red”), “extracellular matrix” (“breast: greenyellow”, “BRCA: brown”), “regulation of transcription” (“breast: turquoise”, “breast: purple”, “BRCA: blue”). More CYT-associated modules were observed. They were themed by “defense response to virus” (“BRCA: salmon”, no corresponding module in breast), “lipid particle” (“BRCA: tan”, corresponding to “breast: yellow”), “structural constituent of ribosome” (“BRCA: magenta”, corresponding to “breast: black”), “chromosome segregation” (“BRCA: yellow”, corresponding to “breast: pink”).


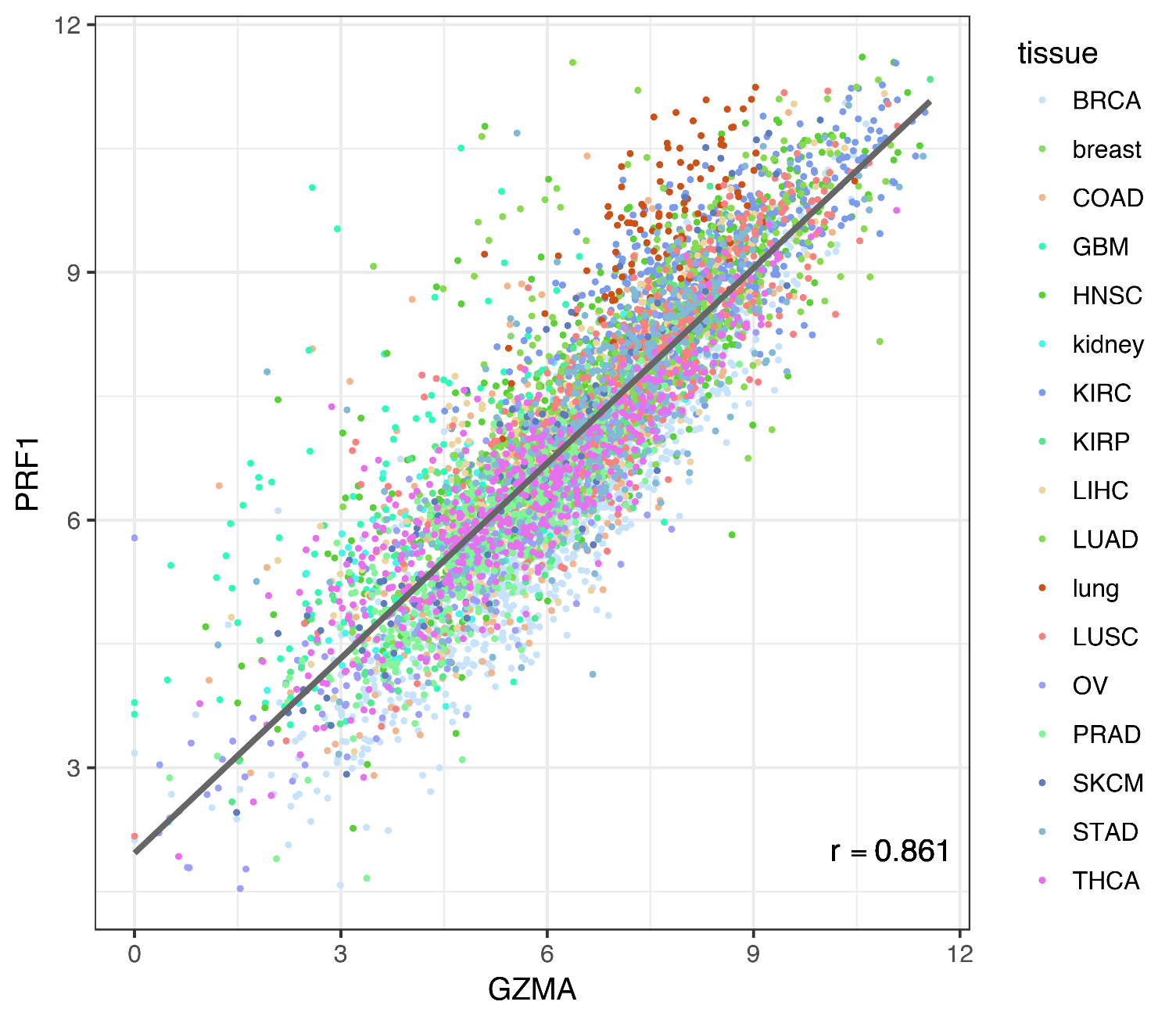


**Supplementary Figure S1.** Correlation (Pearson’s correlation) of expression levels of GZMA and PRF1 across all 6114 samples. Tissues were labeled by colors.

**
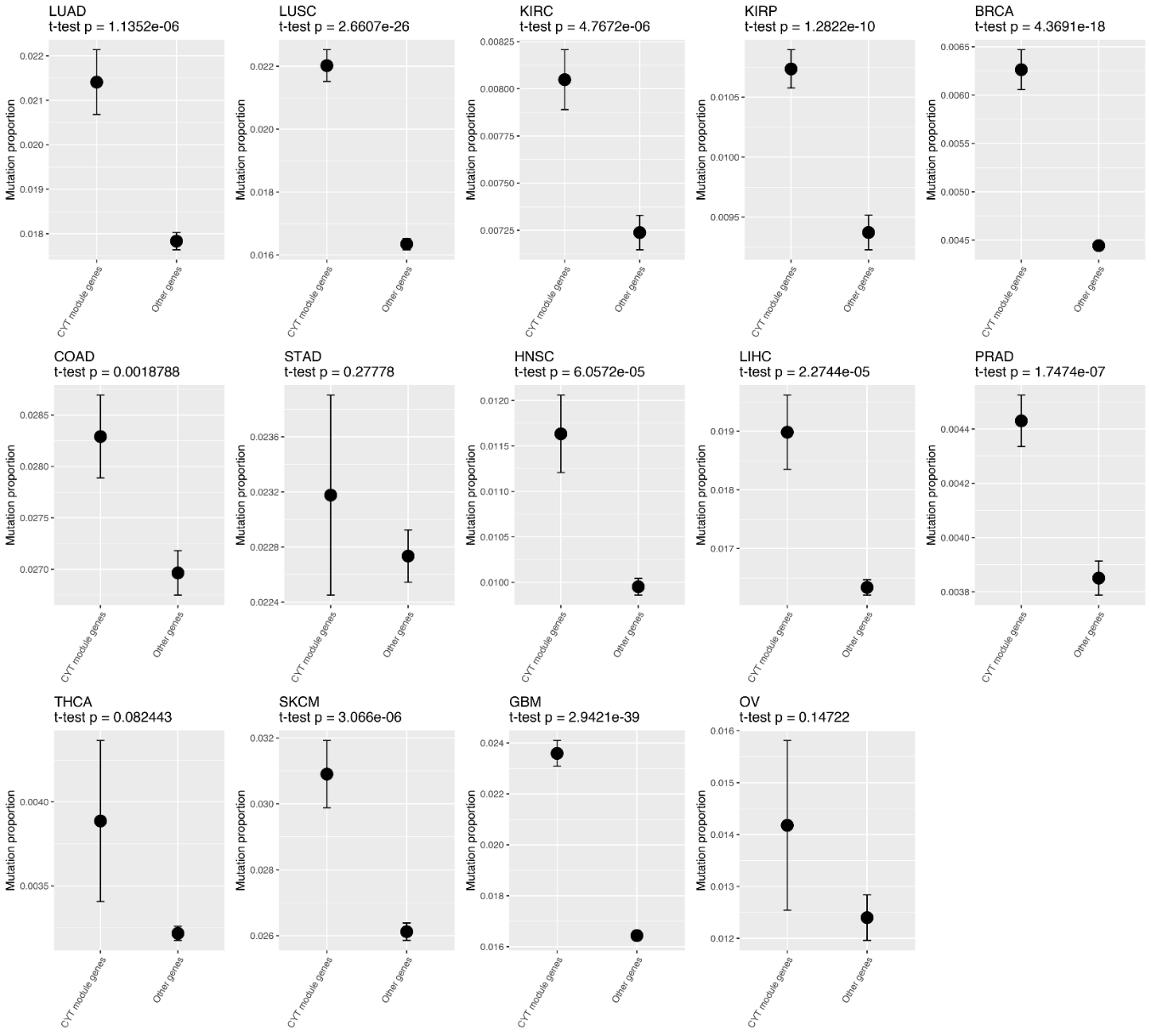
**

**Supplementary SFigure 2.** Mutation burden of genes in CYT-associated modules. The means (the dots) and standard errors (error bars) and mutation proportion are displayed. One sided t-test *p-values* are also showed.

**
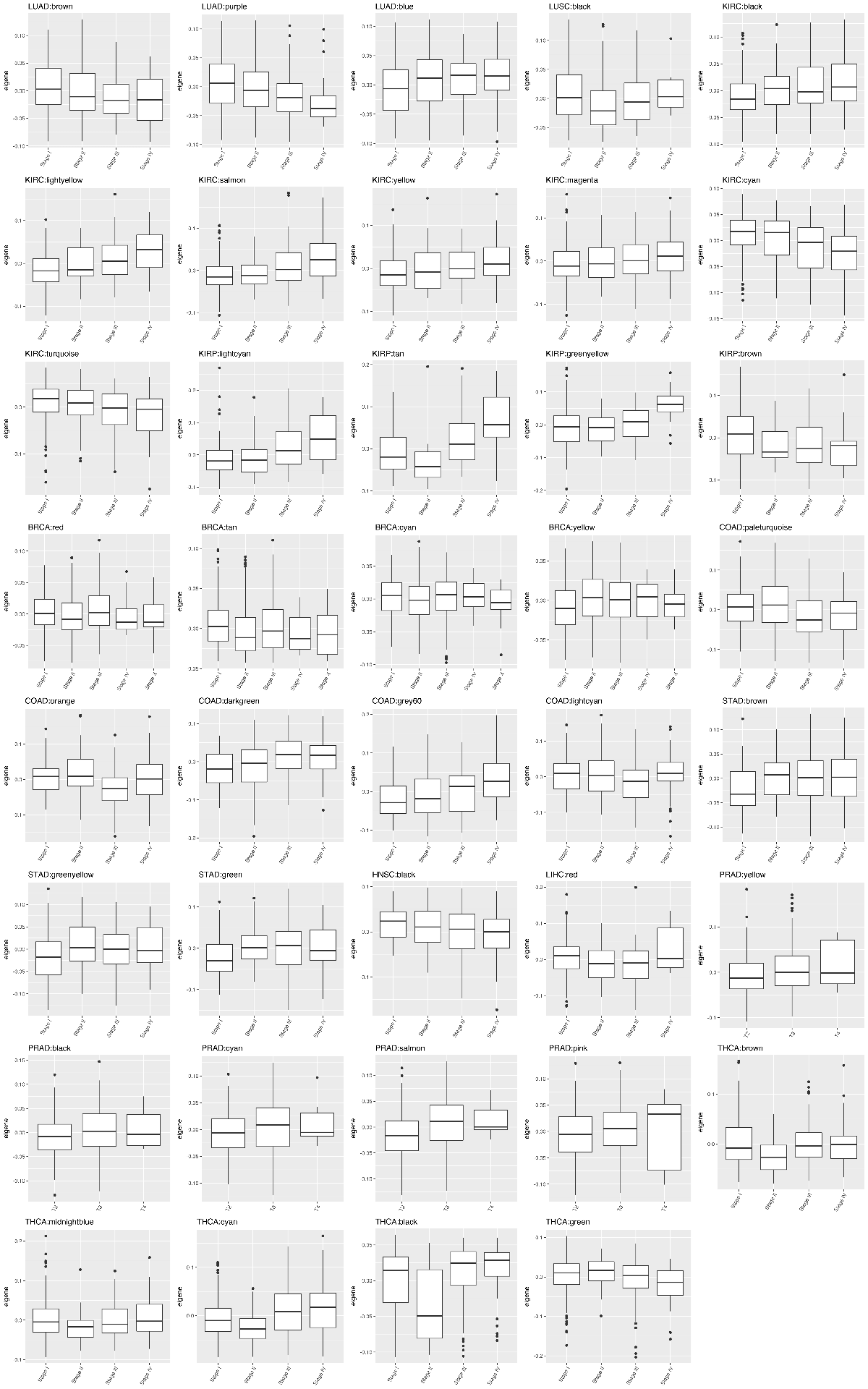
**

**Supplementary Figure S3.** Plots of CYT-associated modules in cancer stages. Correlations between module eigenes and stages were identified by Kruskal-Wallis rank sum test (*p-value* < 0.05).
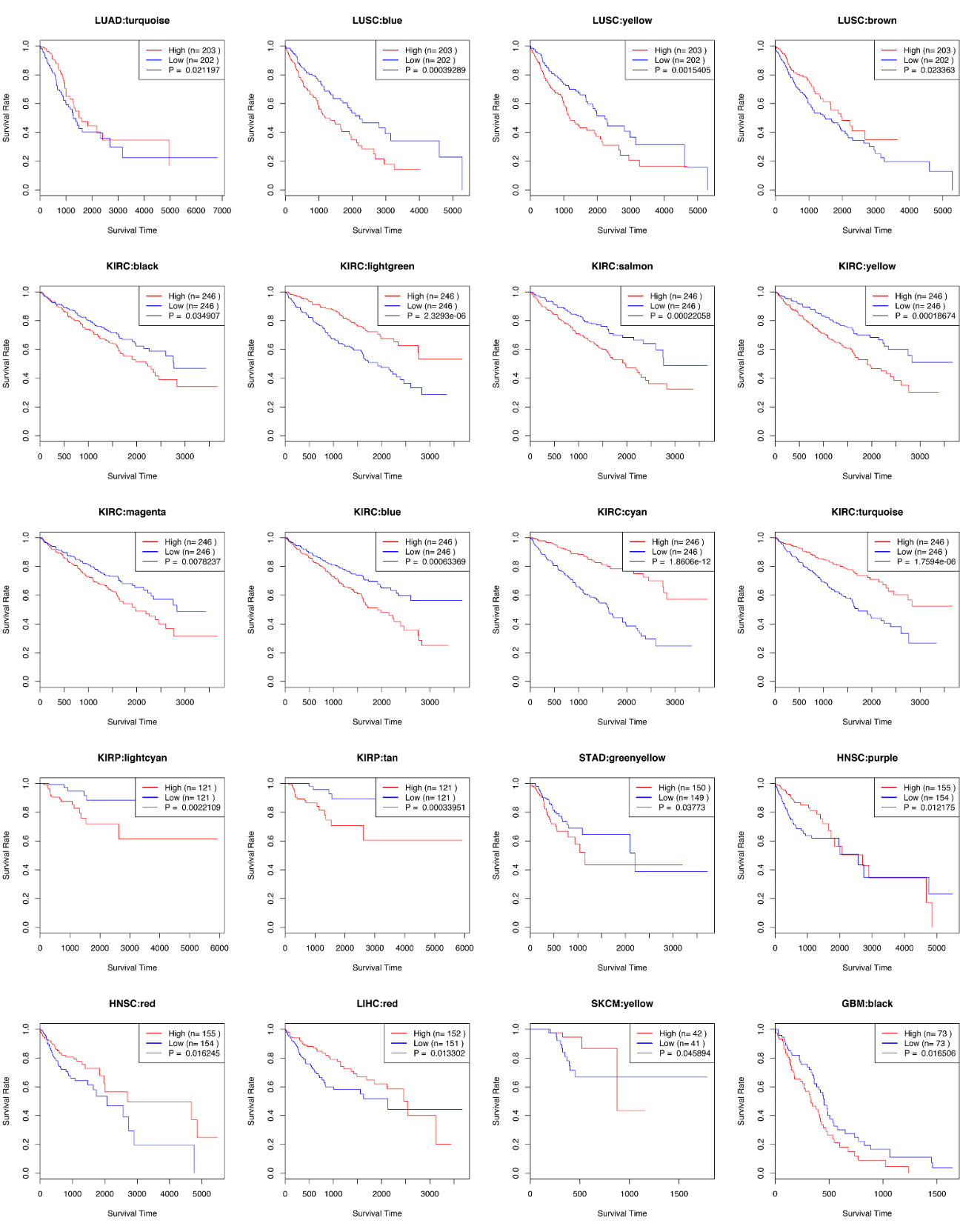


**Supplementary Figure S4.** Plots of survival analysis of CYT-associated modules. High and low groups were defined by median of module eigenes.

**
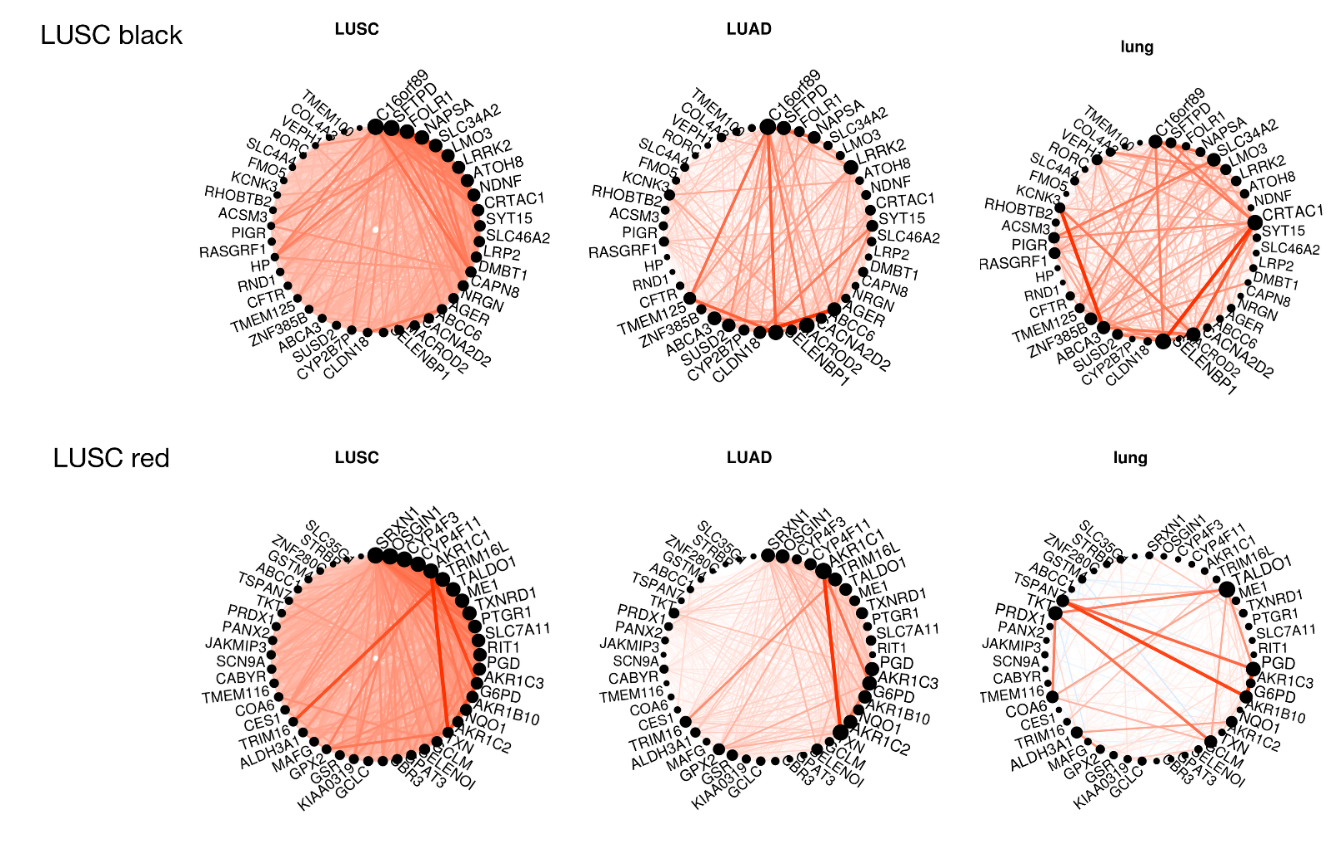
**

**Supplementary Figure S5.** Visualization of “LUSC: black” and “LUSC: red” modules in LUSC, LUAD and lung networks. The thickness of the line reflects the absolute correlation. The line is colored in red if the correlation is positive and green if it is negative. The size of each black circle indicates the connectivity of the corresponding gene; hubs (i.e., highly connected) genes are represented by larger circles. The order of genes is corresponding to the connectivity of genes in “LUSC: black” and “LUSC: red” respectively. Visual inspection suggests these genes are co-expressed in LUSC only.


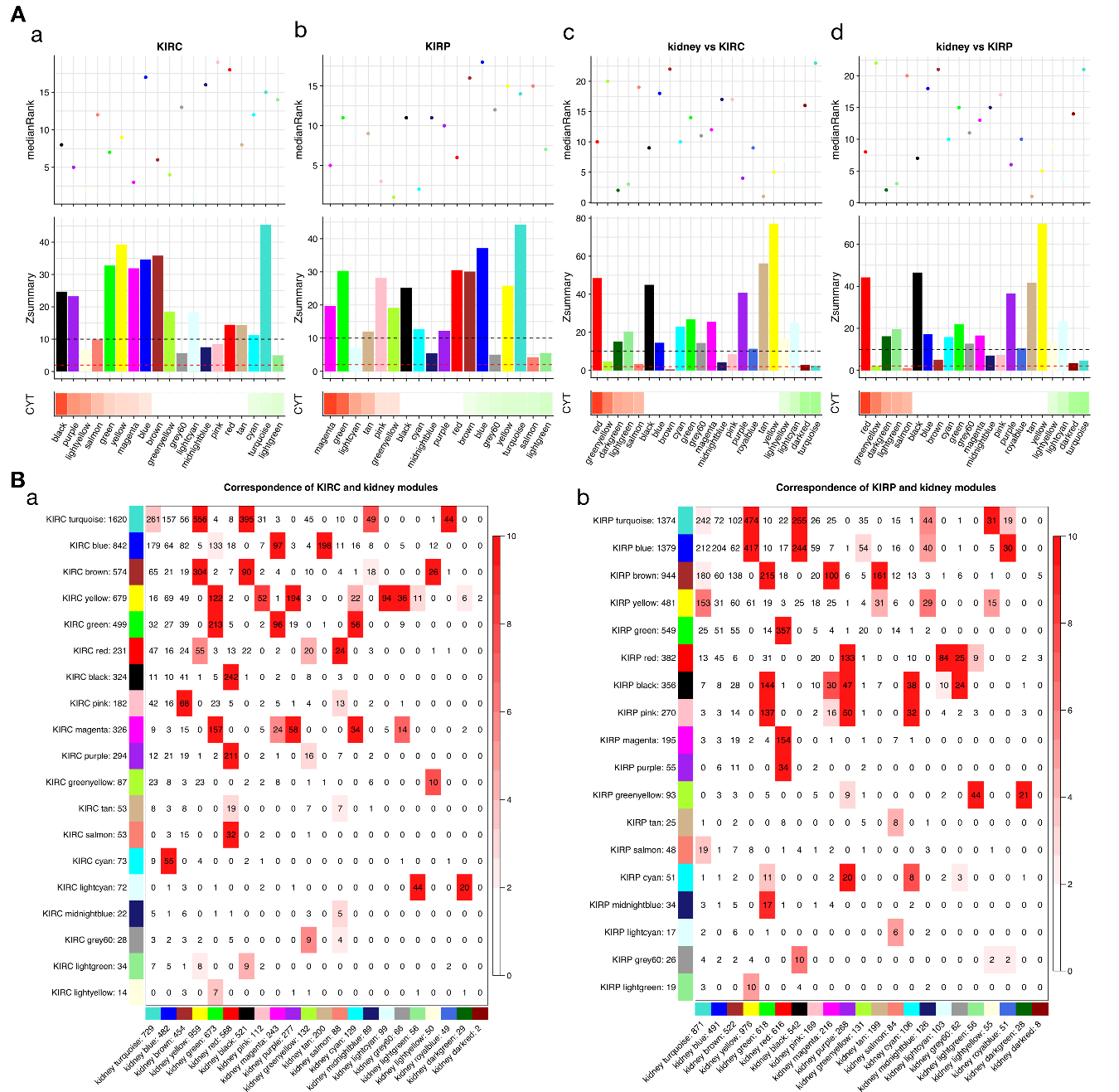


**Supplementary Figure S6.** Visualization of CYT-associations and preservations of modules in KIRC, KIRP and kidney. (**A**) In each panel, below band is a plot of module-CYT correlation, with red indicating positively correlated and green indicating negatively correlated. The bar plot in middle shows Z-summary statistics. Red dashed line indicates 2, black dashed line indicates 10. The dot plot in upper shows medianRank statistics. (a) CYT correlation and preservation in kidney network of KIRC modules. (b) CYT correlation and preservation in kidney network of KIRP modules. (c) CYT correlation and preservation in KIRC network of kidney modules. (d) CYT correlation and preservation in KIRP network of kidney modules. (**B**) Correspondence of (a) KIRC (b) KIRP modules and kidney modules. Numbers in the table indicate gene counts in the intersection of the corresponding modules. Coloring of the table encodes –log(p), with p being the Fisher’s exact test p-value for overlap of two modules.


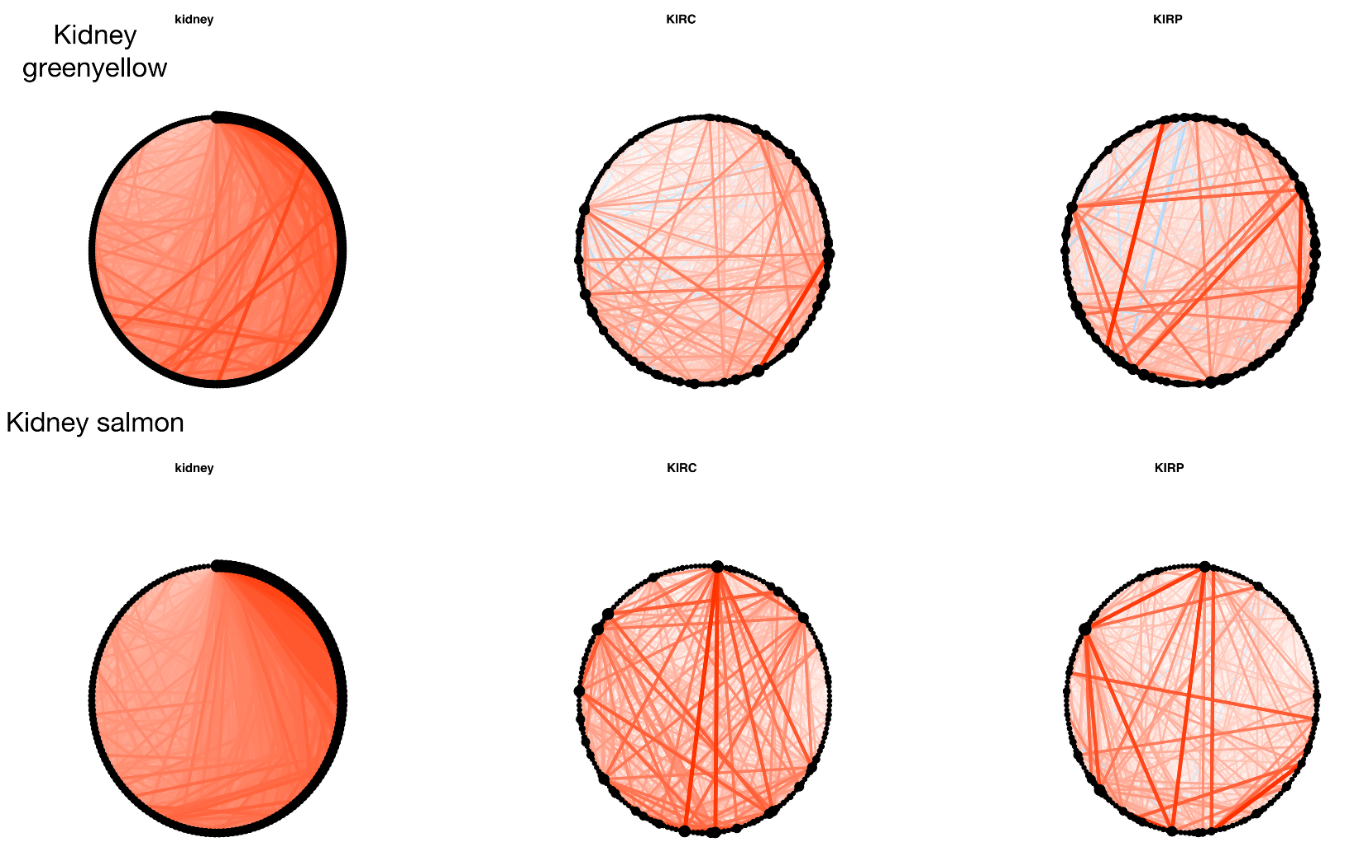


**Supplementary Figure S7.** Visualization of “kidney: greenyellow” and “kidney: salmon” modules in kidney, KIRC and KIRP networks. Visual inspection suggests these genes are co-expressed in kidney only. The order of genes is corresponding to the connectivity of genes in “kidney: greenyellow” and “kidney: salmon” respectively.


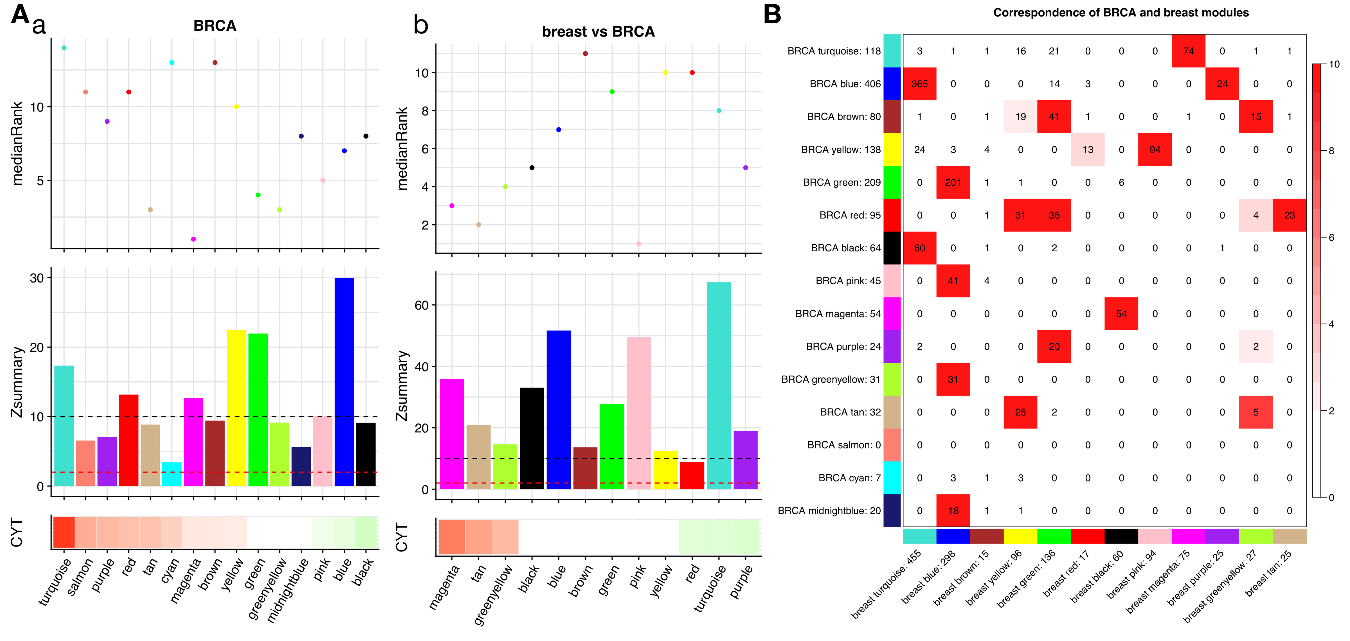


**Supplementary Figure S8.** Visualization of CYT-associations and preservations of modules in BRCA and breast. (**A**) In each panel, below band is a plot of module-CYT correlation, with red indicating positively correlated and green indicating negatively correlated. The bar plot in middle shows Z-summary statistics. Red dashed line indicates 2, black dashed line indicates 10. The dot plot in upper shows medianRank statistics. (a) CYT correlation and preservation in breast network of BRCA modules. (b) CYT correlation and preservation in BRCA network of breast modules. (**B**) Correspondence of BRCA modules and breast modules. Numbers in the table indicate gene counts in the intersection of the corresponding modules. Coloring of the table encodes –log(p), with p being the Fisher’s exact test p-value for overlap of two modules.

**
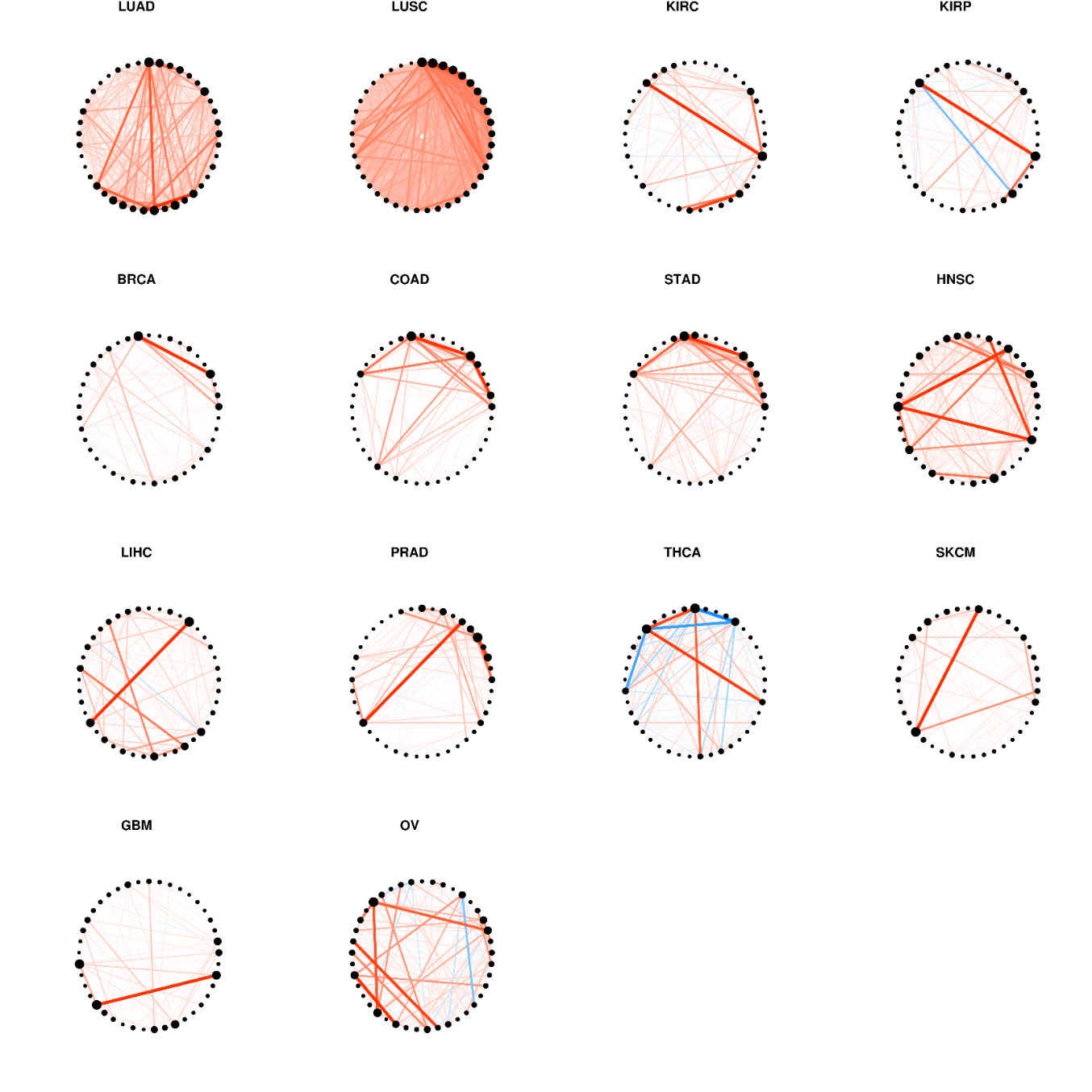
**

**Supplementary Figure S9.** Visualization of “LUSC: black” across 14 tumor networks. The order of genes is corresponding to the connectivity of genes in “LUSC: black”.


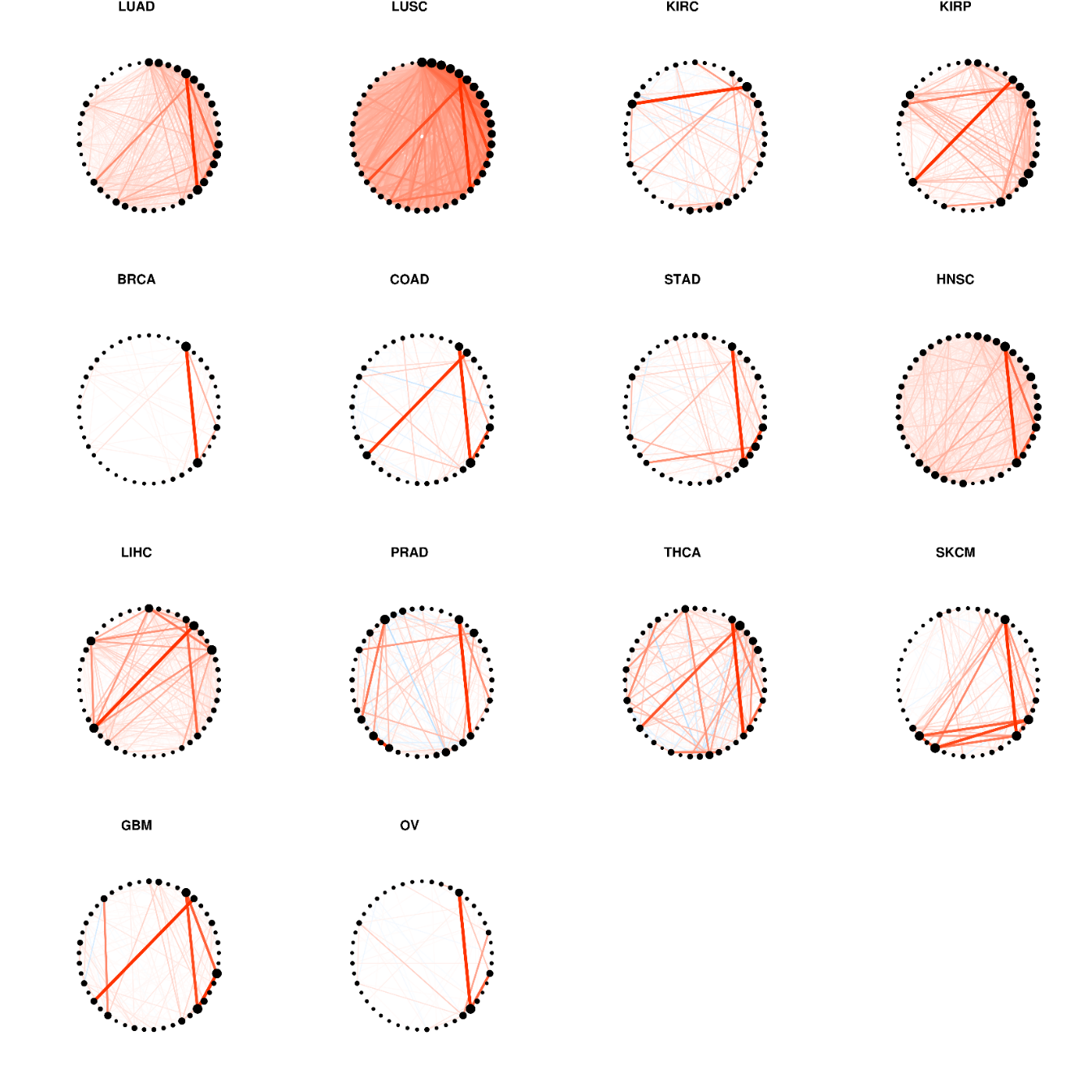


**Supplementary Figure S10.** Visualization of “LUSC: red” across 14 tumor networks. The order of genes is corresponding to the connectivity of genes in “LUSC: red”.


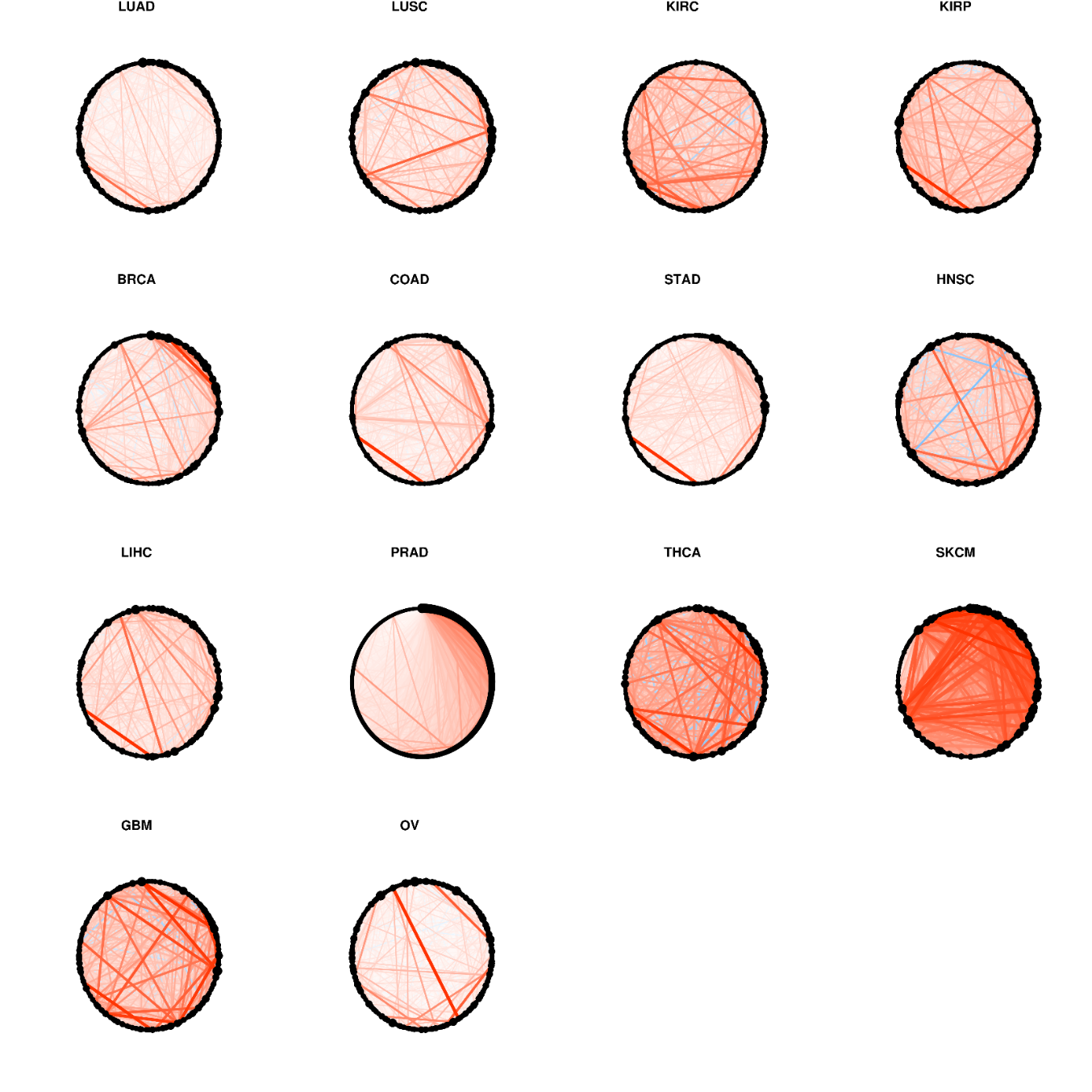


**Supplementary Figure S11.** Visualization of “PRAD: magenta” across 14 tumor networks. The order of genes is corresponding to the connectivity of genes in “PRAD: magenta”.


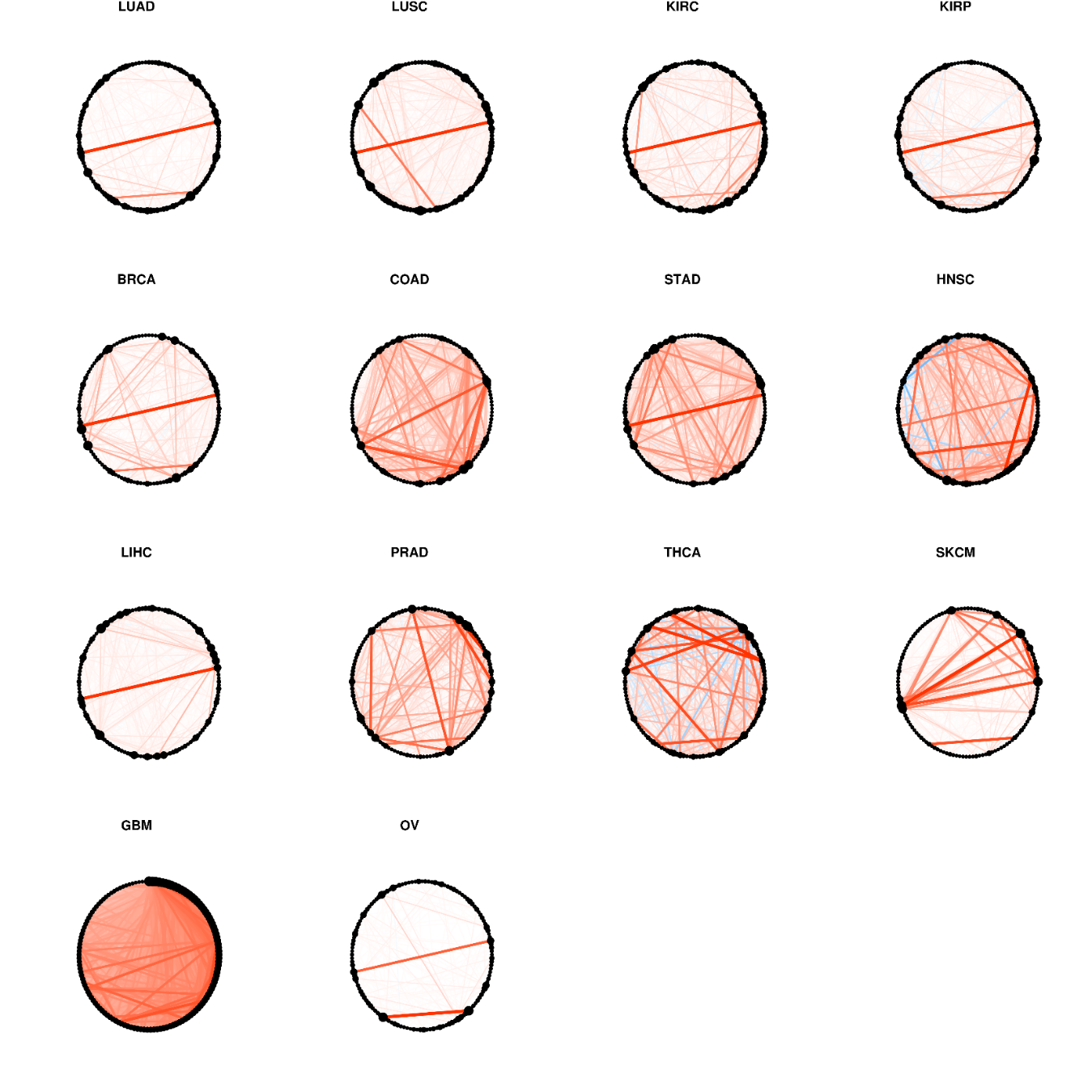


**Supplementary Figure S12.** Visualization of “GBM: black” across 14 tumor networks. The order of genes is corresponding to the connectivity of genes in “GBM: black”.

**Supplementary Table 1.** Summary of TCGA gene expression data used to construct co-expression networks.

| **Tissue Type** | **Age** | | | **Gender** | | **Ethnicity** | | | | **Tumor Location** | | **Sample Size** | |
| --- | --- | --- | --- | --- | --- | --- | --- | --- | --- | --- | --- | --- | --- |
|  | **Mean** | **Median** | **Range** | **Male** | **Female** | **White** | **Hispanic** | **Black** | **Asian** | **Primary** | **Metastatic** | **After Filtering** | **Before Filtering** |
| **LUAD** | 65.58 | 67 | 38 - 88 | 223 | 265 | 367 | 5 | 49 | 8 | 331 | 154 | 488 | 515 |
| **LUSC** | 67.28 | 68 | 39 - 90 | 358 | 121 | 331 | 7 | 29 | 9 | 395 | 80 | 480 | 502 |
| **KIRC** | 60.7 | 61 | 26 - 90 | 324 | 177 | 435 | 25 | 51 | 8 | 396 | 103 | 501 | 533 |
| **KIRP** | 61.36 | 61 | 28 - 88 | 207 | 68 | 197 | 11 | 57 | 5 | 88 | 172 | 275 | 290 |
| **BRCA** | 58.47 | 59 | 26 - 90 | 12 | 1036 | 735 | 37 | 160 | 59 | 868 | 180 | 1049 | 1094 |
| **COAD** | 65.06 | 67 | 31 - 90 | 150 | 120 | 182 | 4 | 53 | 11 | 183 | 84 | 272 | 285 |
| **STAD** | 65.94 | 68 | 30 - 90 | 253 | 138 | 242 | 5 | 12 | 83 | 345 | 46 | 391 | 415 |
| **HNSC** | 60.81 | 61 | 19 - 90 | 365 | 133 | 425 | 24 | 45 | 11 | 176 | 56 | 498 | 520 |
| **LIHC** | 59.25 | 61 | 16 - 90 | 244 | 114 | 178 | 18 | 17 | 151 | 255 | 103 | 358 | 371 |
| **PRAD** | 60.98 | 61.5 | 41 - 77 | 468 | 0 | 143 | 0 | 7 | 2 | 430 | 1 | 468 | 496 |
| **THCA** | 46.98 | 46 | 15 - 89 | 128 | 350 | 315 | 36 | 27 | 51 | 271 | 206 | 478 | 504 |
| **SKCM** | 64.64 | 65 | 24 - 90 | 60 | 39 | 90 | 4 | 0 | 7 | 94 | 3 | 99 | 103 |
| **GBM** | 60.02 | 60.5 | 21 - 89 | 96 | 52 | 134 | 3 | 8 | 5 | NA | NA | 151 | 156 |
| **OV** | 58.85 | 58 | 30 - 87 | 0 | 289 | 241 | 3 | 23 | 12 | NA | NA | 290 | 305 |
| **lung** | 67.46 | 67 | 45 - 86 | 61 | 44 | 93 | 0 | 7 | 0 | 70 | 32 | 105 | 110 |
| **kidney** | 62.71 | 63 | 28 - 90 | 73 | 28 | 88 | 5 | 7 | 0 | 68 | 32 | 101 | 104 |
| **breast** | 57.12 | 56 | 30 - 90 | 1 | 109 | 103 | 0 | 6 | 1 | 101 | 9 | 110 | 113 |

**Supplementary Table 2.** Summary of CYT-associated modules across 14 types of tumors. Columns from left to right are: name of modules (tissue: module color), CYT correlation, FDR of CYT correlation, size of modules, representative enriched GO terms, intersection number of genes in module and representative GO term, enrichment FDR, median of 13 Z-summary (module preservation in other 13 tumor networks), representative GO theme selected by REVIGO, hub genes in modules, Kruskal-Wallis *p-value* for cancer stages and survival *p-value*.

**Supplementary Table 3**

Gene ontology analysis of moderate conserved CYT-associated modules, which are defined as median 2 < Z-summary < 10.

| **Module** | **CYT correlation** | **CYT adjust p** | **Size** | **Representative GO** | **Intersect size** | **Enrichment adjust p** |
| --- | --- | --- | --- | --- | --- | --- |
| KIRC:lightyellow | 0.483655412 | 3.41E-31 | 24 | GO:0008064: regulation of actin polymerization or depolymerization | 6 | 0.006282833 |
| KIRC:cyan | -0.149121774 | 0.001600027 | 106 | GO:0046395: carboxylic acid catabolic process | 16 | 8.63E-08 |
| BRCA:tan | 0.316862948 | 8.28E-26 | 34 | GO:0005811: lipid particle | 6 | 0.0000947 |
| COAD:paleturquoise | 0.10999608 | 0.089019407 | 41 | GO:0005740: mitochondrial envelope | 16 | 5.18E-08 |
| COAD:skyblue | -0.182054336 | 0.0036052 | 48 | GO:0031981: nuclear lumen | 30 | 0.0000292 |
| COAD:darkgreen | -0.234512576 | 0.000173967 | 61 | GO:0005654: nucleoplasm | 28 | 0.020641065 |
| COAD:skyblue3 | -0.270245014 | 0.0000161 | 31 | GO:0001047: core promoter binding | 6 | 0.023902891 |
| COAD:midnightblue | -0.31180448 | 0.000000522 | 95 | GO:0044444: cytoplasmic part | 71 | 0.033757548 |
| THCA:black | 0.196557728 | 0.0000366 | 403 | GO:0071944: cell periphery | 179 | 9.91E-12 |
| SKCM:red | 0.380352671 | 0.00066823 | 405 | GO:0006954: inflammatory response | 41 | 0.0000616 |
| GBM:cyan | 0.167981281 | 0.049766952 | 45 | GO:0044782: cilium organization | 17 | < 2.2E-16 |
| GBM:magenta | -0.416709752 | 0.00000027 | 94 | GO:0005654: nucleoplasm | 58 | < 2.2E-16 |

**Supplementary Table 4**

WGCNA parameters

| **Parameters** | **Value** |
| --- | --- |
| maxPOutlier | 0.1 |
| scale-free topology fitting index | 0.8 |
| height cutoff | 0.95 |
| deepSplit | 4 |
| minimum cluster size | 20 |
